# IRAP-dependent endosomal T cell receptor signalling is essential for T cell responses

**DOI:** 10.1038/s41467-020-16471-7

**Published:** 2020-06-02

**Authors:** Irini Evnouchidou, Pascal Chappert, Samira Benadda, Andres Zucchetti, Mirjana Weimershaus, Marcelle Bens, Vivien Caillens, Despoina Koumantou, Sophie Lotersztajn, Peter van Endert, Jean Davoust, Pierre Guermonprez, Claire Hivroz, David A. Gross, Loredana Saveanu

**Affiliations:** 1 Université de Paris, Centre de recherche sur l’inflammation, INSERM U1149, CNRS ERL8252, 75018 Paris, France; 2 Inovarion, 75005 Paris, France; 3 Université de Paris, Institut Necker Enfants Malades, INSERM U1151, CNRS U8253, 75015 Paris, France; 4 Paris Sciences and Lettres Research University, Institut Curie, INSERM U932, 75005 Paris, France; 5 Université Paris-Saclay, UVSQ, Inserm, END-ICAP, 78000 Versailles, France; 6 Centre for Inflammation Biology and Cancer Immunology, King’s College London, SE1 1UL, London, UK; 7 Integare, UMR_S951, Genethon, Inserm, Univ Evry, Université Paris-Saclay, Evry F91000, Paris, France

**Keywords:** Cell signalling, Lymphocyte activation, T cells, Signal transduction

## Abstract

T cell receptor (TCR) activation is modulated by mechanisms such as TCR endocytosis, which is thought to terminate TCR signalling. Here we show that, upon internalization, TCR continues to signal from a set of specialized endosomes that are crucial for T cell functions. Mechanistically, TCR ligation leads to clathrin-mediated internalization of the TCR-CD3*ζ* complex, while maintaining CD3*ζ* signalling, in endosomal vesicles that contain the insulin responsive aminopeptidase (IRAP) and the SNARE protein Syntaxin 6. Destabilization of this compartment through IRAP deletion enhances plasma membrane expression of the TCR-CD3*ζ* complex, yet compromises overall CD3*ζ* signalling; moreover, the integrity of this compartment is also crucial for T cell activation and survival after suboptimal TCR activation, as mice engineered with a T cell-specific deletion of IRAP fail to develop efficient polyclonal anti-tumour responses. Our results thus reveal a previously unappreciated function of IRAP-dependent endosomal TCR signalling in T cell activation.

## Abstract (web summary)

T cell receptors (TCR) are internalized when activated by their ligands. Here the authors show that the internalized TCRs are localized to endosomes expressing IRAP and Syntaxin 6 to maintain intracellular signalling capacity, whose importance is shown by the absence of efficient polyclonal anti-tumour response in mice with T-specific conditional deletion of IRAP.

## Introduction

At the plasma membrane, the antigen T-cell receptor (TCR) complex comprises the clonotypic αβ heterodimers, the associated CD3ε, γ*,* δ chains and a ζ chain dimer, which is commonly named CD3ζ. This multi-subunit antigen receptor recognises a wide variety of cognate peptide–MHC complexes (pMHC) through the variable domain of the TCRα and β chains with different outcomes. Low-affinity recognition of self-pMHC gears T-cell selection in the thymus, T-cell export and T-cell survival in the periphery, while higher-affinity recognition of foreign pMHC initiates effector T-cell responses^1,2^. These processes depend on TCR signalling that is subtly modulated by the regulated trafficking of TCR components, signalling adaptors such as the linker for activation of T cells (LAT) and signalling effectors such as the lymphocyte-specific protein tyrosine kinase (Lck). Upon TCR activation by pMHC, the intracellular TCR signalling components translocate to the plasma membrane from separate vesicular pools: Lck translocates from Rab11^+^ endosomes^3^ and LAT from Rab27a^+^ and VAMP7^+^ endosomes^3–5^. The α, β, ε, γ and δ chains, which have together four immunoreceptor tyrosine-based activation motifs (ITAMs), are mainly located in the endoplasmic reticulum^6,7^. The ζ chain, which is encoded by the *CD247* gene, bears six of the ten ITAMs of the full TCR complex, and is present in distinct vesicles that have not been entirely characterised^3,5,8^.

Better characterisation of this intracellular pool of CD3ζ can help to delineate the mechanisms by which ζ chain expression controls TCR cell surface levels^5,9^. Thus, in the absence of the CD3ζ chain, TCRαβ, CD3γε and CD3δε dimers can associate in the endoplasmic reticulum and can reach the plasma membrane, but their cell surface level is extremely low^5,10^. Mice deficient for the ζ chain have barely detectable TCR expression and show severe defects in T-cell development. Interestingly, in ζ chain-deficient mice, T-cell development can be partially rescued by a signalling incompetent mutant of the ζ chain^11^ that also normalises TCR expression levels at the plasma membrane. The multiple ITAMs of the ζ chain are, however, crucial for T-cell activation in the periphery, as CD3ζ ITAMs were shown to amplify TCR signalling under suboptimal TCR triggering^10,12^.

In this study, we characterise the intracellular localisation and intracellular signalling capacity of the ζ chain of the TCR. We find that, in Jurkat T cells as well as in primary mouse T cells, the ζ chain is localised in an intracellular pool of vesicles described by the Insulin Responsive AminoPeptidase (IRAP) and by the SNARE Syntaxin 6 (Stx6). We show that IRAP interacts with the TCRζ chain and that after TCR engagement, CD3ζ continues to signal from the IRAP^+^ intracellular pool. Destabilization of this compartment through IRAP deletion increases plasma membrane expression of the TCR, but compromises TCR signalling. Consequently, mice harbouring a T-cell-specific deletion of IRAP fail to respond to suboptimal antigen stimulation, and are unable to control the growth of model tumours. Our results demonstrate that the TCR uses endosomal signalling platforms that contribute to peripheral T-cell survival and are essential for anti-tumour T-cell responses.

## Results

### The CD3ζ intracellular pool colocalizes with IRAP and Stx6

**Fig. 1:**
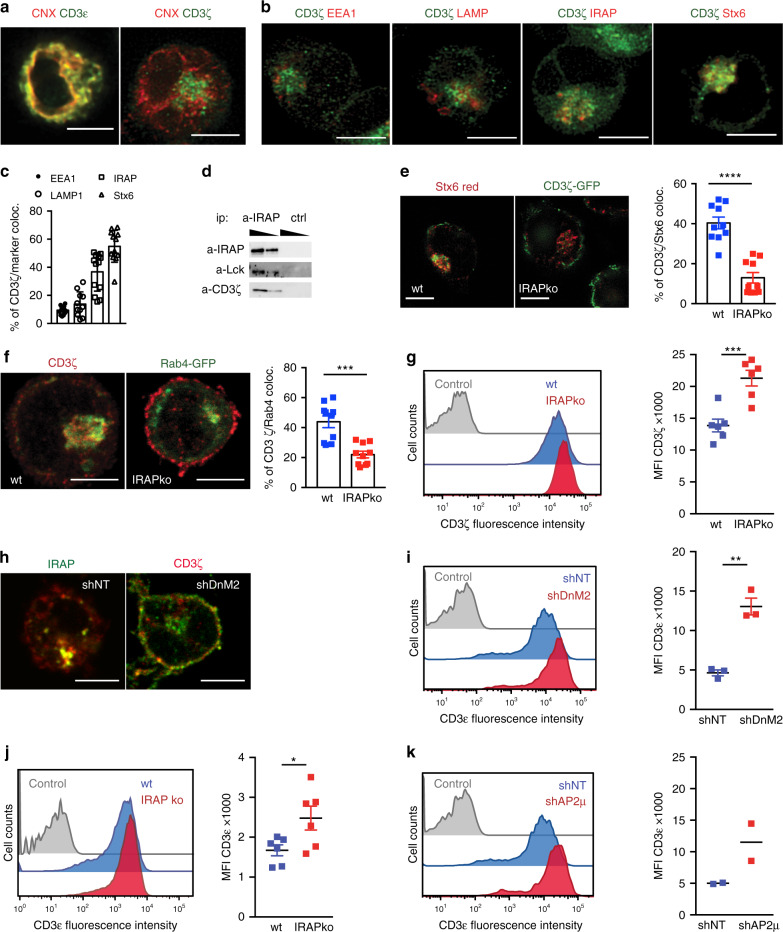
The CD3ζ intracellular pool colocalizes with IRAP and Stx6. **a**, **b**, **e, f, h** Representative images of Jurkat T cells allowed to adhere on poly-L-lysine slides. Bars represent 5-μm scale. Jurkat T cells were fixed and stained for CD3ε or CD3ζ (green) and CNX (red) (**a**), CD3ζ (green) and EEA1 or LAMP1 or IRAP or Stx6 (red) (**b**). **c** Quantification of early and late endosomal marker colocalization with CD3ζ (*n* = 13 cells for EEA1 and IRAP; *n* = 11 for LAMP1 and Stx6). **d** Immunoblot analysis of IRAP, Lck and CD3ζ after immunoprecipitation with anti-IRAP or an isotype control in Jurkat T cells. **e**, **f** CD3ζ colocalization with Stx6 (*n* = 10 cells, ****p < 0.0001) (**e**) and Rab4 (*n* = 10 cells, ***p = 0.0001) (**f**) in wt or IRAP ko Jurkat T cells transduced with a CD3ζ-GFP reporter (**e**) or a Rab4-GFP reporter (**f**). Bars represent 5-μm scale. **g** CD3ζ expression on the cell surface of wt or IRAP ko Jurkat T cells expressing a CD8-CD3ζ reporter, measured by flow cytometry (*n* = 6 experiments, ***p = 0.0008). **h** CD3ζ (red) colocalization with IRAP (green) in wt or DnM2-deficient Jurkat T cells. Bars represent 5 μm scale. **i** CD3ε expression on the cell surface of wt or DnM2-deficient Jurkat T cells, measured by flow cytometry (*n* = 3 experiments, **p = 0.0017). **j** CD3ε expression on the cell surface of wt or IRAP ko Jurkat T cells, measured by flow cytometry (*n* = 6 experiments, *p = 0.0340). **k** CD3ε expression on the cell surface of wt or AP2μ-deficient Jurkat T cells, measured by flow cytometry (*n* = 2 experiments). All *p*-values (**e**, **f**, **g**, **i**, **j**) were calculated with two-tailed unpaired *t* tests. Each symbol represents an individual cell (**c**, **e**, **f**) or an independent experiment (**g**, **i**, **j**). Values represent mean ± SEM. For additional information, see Supplementary Fig. 1.

To investigate the nature of the intracellular ζ pool, we stained Jurkat T cells with markers of the ER (Calnexin, CNX), early endosomes (Rab4, EEA1), late endosomes (LAMP1), storage endosomes (IRAP and Stx6)^13^ and trans-Golgi-derived vesicles (Stx6) and found that the ζ chain colocalizes with IRAP, Stx6 and Rab4 (Fig. 1a–c; Supplementary Fig. 1a, b). We validated ζ chain colocalization with IRAP by co-immunoprecipitation experiments and observed that the first player of TCR signalling, the Src kinase Lck was also co-immunoprecipitated with IRAP (Fig. 1d). Since IRAP affects the trafficking of Stx6 trans-Golgi-derived endosomes^14^, we inactivated IRAP gene expression by both shRNA and CrispR/Cas9 methods (Supplementary Fig. 1c, d) and investigated CD3ζ localisation. In the absence of IRAP, CD3ζ cellular distribution was altered, with a significant decrease in Stx6 and Rab4-associated vesicular pool and an accumulation of CD3ζ at the cell surface (Fig. 1e–g). To determine if the ζ chain is targeted to IRAP/Stx6^+^ vesicles via the secretory pathway or from the cell surface, we screened for molecules possibly involved in ζ chain and IRAP endocytosis. We found that CD3ζ and IRAP were constitutively transported to the cell surface, but were internalized in IRAP-dependent endosomes characterised by the Stx6 marker via an endocytosis pathway that depended on the clathrin adaptor AP2 and DnM2 (Fig. 1h; Supplementary Fig. 1e–h). We observed that, in the absence of IRAP, CD3ζ accumulated at the plasma membrane and increased cell surface expression of CD3ε and TCR complex (Fig. 1i–k). This finding is in agreement with the crucial role of CD3ζ as a limiting component of TCR cell surface expression^9^. Altogether these data identify IRAP as a key controller of CD3ζ and TCR assembly.

### IRAP is required for proper TCR signalling in Jurkat cells

**Fig. 2:**
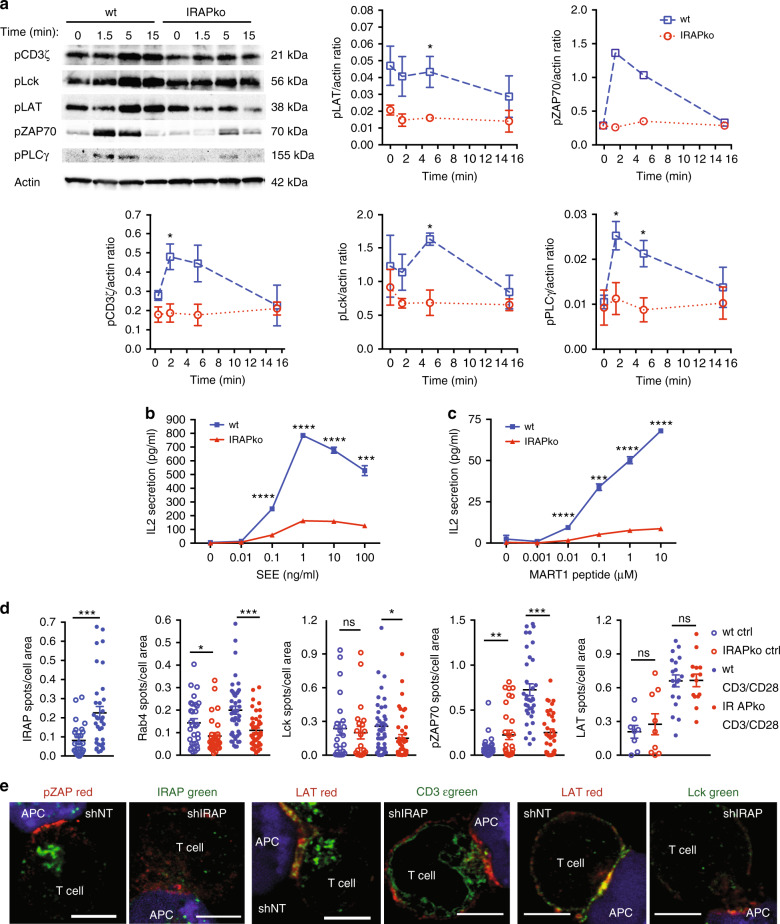
IRAP is required for proper TCR signalling and activation of Jurkat T cells. **a** Immunoblot analysis of TCR signalling molecules in wt and IRAP ko Jurkat T cells after activation by anti-CD3ε/CD28 for various time points. Values represent mean ± SEM of pool of three independent experiments for all signalling components excepting pPLCγ (*n* = 4) and pZAP70 (*n* = 2). pLAT *p = 0.0429, pCD3ζ *p = 0.0231, pLck *p = 0.0107, pPLCγ *p_1.5 min_ = 0.0262, *p_5 min_ = 0.0199. **b**, **c** IL-2 response of Jurkat T cells measured by ELISA: **b** wt or IRAP ko Jurkat T cells were incubated for 6 h with Raji B cells presenting the SEE superantigen, **c** wt or IRAP ko Jurkat T cells expressing the MART1 TCR were incubated overnight with Daju-A2 cells presenting the MART1 peptide. Values represent mean ± SEM of three replicates of one representative experiment of three independent experiments. SEE ****p < 0.0001, ***p = 0.0004, MART1 ****p < 0.0001, ***p = 0.0002. **d** Quantification of TIRF microscopy of IRAP, Rab4, Lck, pZAP70 and LAT membrane recruitment in CD3ε/CD28-activated Jurkat T cells. Wt or IRAP ko Jurkat T cells were activated (CD3/CD28) or not (Ctrl) for 10 min on CD3ε/CD28-coated slides, fixed and stained for IRAP or pZAP70 or LAT. For Rab4 and Lck, cells were transfected with Rab4-GFP and Lck-GFP, respectively. Each dot represents a cell (IRAP: wtCtrl *n* = 28, wtCD3/CD28 *n* = 34, ***p = 0.0005; Rab4: wtCtrl *n* = 30, wtCD3/CD28 *n* = 46, IRAPkoCtrl *n* = 30, IRAPkoCD3/CD28 *n* = 45, *p = 0.016, ***p = 0.0001; Lck wtCtrl *n* = 23, wtCD3/CD28 *n* = 41, IRAPkoCtrl *n* = 21, IRAPkoCD3/CD28 *n* = 42, *p = 0.033; pZAP70 wtCtrl *n* = 33, wtCD3/CD28 *n* = 35, IRAPkoCtrl *n* = 31, IRAPkoCD3/CD28 *n* = 34, **p = 0.009, ***p = 0.0001; LAT wtCtrl *n* = 8, wtCD3/CD28 *n* = 19, IRAPkoCtrl *n* = 9, IRAPkoCD3/CD28 *n* = 14). Values represent mean ± SEM. All *p*-values (**a**, **b**, **c**, **d**) were calculated with two-tailed unpaired *t* tests. **e** Confocal microscopy of SEE-pulsed Raji B cell/Jurkat T-cell conjugates. Cells were stained for: Lat (red), Lck (green)—right panel, Lat (red), CD3ε (green)—middle panel and pZAP70 (red), IRAP (green)—left panel. Bars represent 5-μm scale. Images are representative of three independent experiments. For additional information, see Supplementary Fig. 2.

Since increased TCR expression at the plasma membrane was previously associated with an amplified TCR function^15^, we investigated whether TCR signalling was enhanced in IRAP-deficient Jurkat T cells. Unexpectedly, TCR signalling was decreased, as shown by reduced phosphorylation of several TCR signalling partners, such as Lck, LAT, ZAP-70, PLCγ and CD3ζ itself (Fig. 2a; Supplementary Fig. 2a). Moreover, IRAP-deficient T cells displayed a strong defect in IL-2 secretion after stimulation with the staphylococcal enterotoxin E (SEE) superantigen loaded on Raji B cells (Fig. 2b). Similar defects in IL-2 production were observed when IRAP-deficient cells expressing the MART1 TCR^16^ were stimulated with the cognate peptide bound to the HLA-A2 complex (Fig. 2c).

Since IRAP deficiency leads to increased TCR accumulation at the cell surface, these results demonstrated that there is no direct correlation between TCR cell surface expression levels and TCR signalling intensity. Therefore, we hypothesised that the defective TCR signalling observed in IRAP-deficient cells could be attributed to CD3ζ depletion from the intracellular pools (Fig. 1e, f). The intracellular pool of CD3ζ is known to be polarised and recruited to the immune synapse (IS) to consolidate it^17^. Besides, intracellular CD3ζ might also be required as an intracellular signalling platform for amplifying TCR signalling^18,19^.

To discriminate between these possibilities, we first investigated if IRAP, which colocalized with the CD3ζ pool (Fig. 1b, h), is recruited to the IS. For this, we activated Jurkat T cells using plate-bound anti-CD3ε/anti-CD28 antibodies as a surrogate of antigen presenting cells (APC) and investigated IS dynamics by total internal reflection fluorescence (TIRF) microscopy. IRAP was indeed recruited to the IS (Fig. 2d). IRAP-deficient cells displayed lower recruitment of Rab4 and key signalling proteins, such as Lck and pZAP-70 at the IS. By contrast, the recruitment of the adaptor LAT (Fig. 2d) was unaffected. We conclude that, IRAP deficiency perturbed anti-CD3ε/anti-CD28 TCR signalling events. However, due to the presence of anti-CD3ε antibodies on the plate, the TIRF assay cannot be used to monitor TCR polarisation to the IS. In order to assess the effect of IRAP deficiency on TCR polarisation, we analysed IS formation in T-cell–APC conjugates and, in agreement with the TIRF results, observed low levels of TCR, Lck and pZAP-70, but normal levels of LAT at the IS of IRAP-deficient cells (Fig. 2e; Supplementary Fig. 2b).

In addition to new supply from the TCR intracellular pools, the signalling at the IS is also modulated by TCR internalization^18^. We thus monitored the endocytosis of activated TCR in IRAP-deficient cells. We found that both CD3ε and a CD3ζ reporter containing a CD8-tag were similarly internalized in wt and IRAP-deficient cells (Supplementary Fig. 2c). Activated TCR has been shown to be recycled after internalization^17,20^. However, while the CD3ε subunit was also found to recycle, CD3ζ did not exhibit such behaviour in Jurkat T cells (Supplementary Fig. 2d). Thus, apart from the distinct intracellular localisation at steady state, there is a fundamental difference between the intracellular trafficking of CD3ζ and CD3ε. Since CD3ζ and CD3ε endocytosis were similar in wt and IRAP-deficient cells upon TCR activation, the lower recruitment of CD3ε and signalling molecules at the IS of IRAP-deficient cells likely results from a defect in CD3ζ supply from the intracellular pool, which is strongly reduced in IRAP-deficient cells. In sum, we conclude that IRAP controls the intracellular pool of CD3ζ required for TCR signalosome assembly.

### The TCR is able to signal from IRAP/Stx6^+^ endosomes

**Fig. 3:**
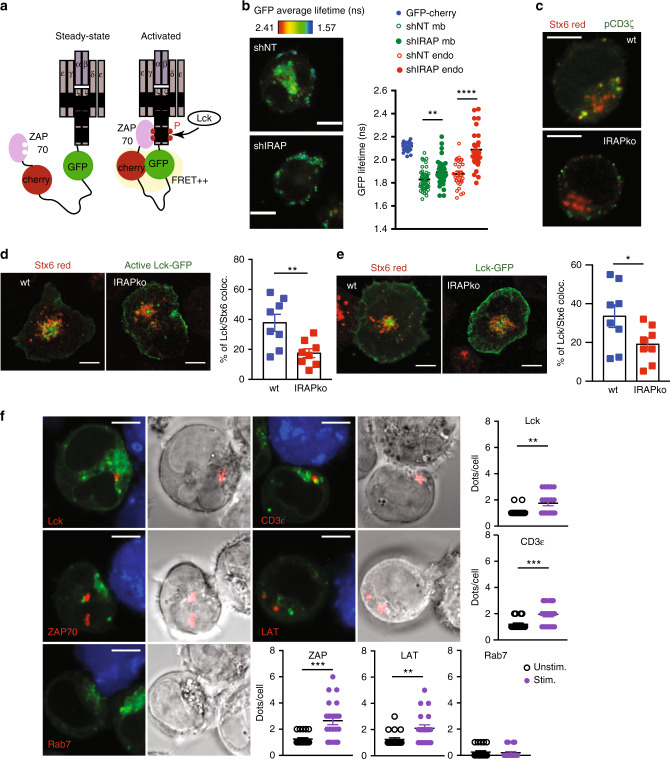
The TCR signals from IRAP/Stx6+ intracellular compartments. **a** CD3ζ reporter composed of a CD3ζ chain followed by a GFP molecule, an mCherry molecule and an SH2 domain of ZAP-70. At the activated state, Lck phosphorylates the CD3ζ ITAMs, which then recruit the SH2 domain of ZAP-70. Then GFP and mCherry come to a distance where FRET can occur. **b** Representative images and quantification of GFP average lifetime in CD3ε/CD28-activated Jurkat T cells. Jurkat T cells expressing the CD3ζ reporter and transduced with either shNT or shIRAP lentivirus were activated on CD3ε/CD28-coated slides for 10 min, fixed and GFP average lifetime was measured by FRET–FLIM. Green symbols represent quantification at the plasma membrane (mb), and red symbols quantification in endosomes (endo). Each dot represents an individual cell (GFP *n* = 46; GFP-mCherry *n* = 38; wt mb *n* = 43; ko mb *n* = 39; wt endo *n* = 28; ko endo *n* = 30, **p = 0.0064, ****p < 0.0001). Bars represent 5-μm scale**. c** Wt or IRAP ko Jurkat T cells were activated on CD3ε/CD28-coated slides for 10 min and stained with anti- CD3ζ pY142 and anti-Stx6 antibodies. Bars represent 5-μm scale**. d**, **e** Colocalization of Stx6 (red) with Lck (green) in wt or IRAP ko Jurkat T cells transfected with Lck(Y505F)-GFP (**d**, *n* = 8 wt, *n* = 8 IRAP ko cells, **p = 0.0070) or Lck-GFP (**e**, *n* = 8 wt, *n* = 8 IRAP ko cells, *p = 0.0486). Bars represent 5-μm scale**. f** Duolink proximity ligation assay of IRAP with various signalling proteins on conjugates formed by Raji B cells presenting SEE superantigen to wt Jurkat T cells expressing Rab4-GFP. Interactions of less than 40-nm distance between the two proteins appear as a red dot. Zoom, original magnification × 4. Bars represent 5 μm scale. At least 20 cells per condition were quantified. Lck **p = 0.0027, CD3ε ***p = 0.0008, ZAP ***p = 0.0001, LAT **p = 0.0054. Each dot represents an individual cell, and all values represent mean ± SEM (**b**, **d, e, f**). All *p*-values were calculated with two-tailed unpaired *t* tests. For additional information, see Supplementary Fig. 3.

Along with providing new molecules for the IS, we hypothesised that the endosomal pool of CD3ζ could serve for sustained signalling, in accordance with the defective T-cell activation observed in the absence of the major endocytosis factor, DnM2^19^. To investigate this hypothesis, we took advantage of the TCR signalling reporter system based on FRET–FLIM using a CD3ζ reporter molecule developed by Yudushkin et al.^18^. This reporter contains a GFP and an mCherry protein flanked by the CD3ζ chain and an SH2 domain of ZAP-70 (Fig. 3a). The CD3ζ reporter showed the same intracellular distribution as the endogenous CD3ζ, and its expression was similarly increased at the cell surface in IRAP-deficient cells (Supplementary Fig. 3a). To test ZAP-70 binding to phosphorylated ITAMs of the CD3ζ reporter, we activated the cells by plate-bound anti-CD3ε/anti-CD28 antibodies and measured the lifetime of GFP by FRET–FLIM. While in control cells the FRET–FLIM signal was present both at the plasma membrane and in an intracellular pool, in IRAP-deficient cells, the FRET–FLIM signal was diminished, presenting a much stronger reduction in the intracellular pool (Fig. 3b; Supplementary Fig. 3b), indicating a predominantly intracellular signalling defect. Moreover, using the CD3ζ reporter, we visualised IS formation in live cells and we observed that in wt cells the IS is very dynamic, getting constant supply of CD3ζ from the intracellular pool, while in IRAP-deficient cells the IS is rather static and supplied with CD3ζ almost exclusively from the plasma membrane (Supplementary Movies 1–4). Similar to the FRET–FLIM reporter, the phosphorylated form of endogenous CD3ζ was mainly intracellular in wt cells, and exclusively located at the plasma membrane in IRAP-deficient cells after cell activation (Fig. 3c). Lck activity is a pre-requisite for the phosphorylation of CD3ζ ITAMs and CD3ζ reporter activation. Identical to the FRET signal and the phosphorylated CD3ζ distribution, a constitutively active form of Lck, as well as wt Lck, were localised both at the plasma membrane and in intracellular Stx6^+^ vesicles in activated wt cells, while in activated IRAP-deficient cells they localised predominantly to the plasma membrane (Fig. 3d, e).

To confirm effective TCR signalling in IRAP intracellular vesicles, we performed a proximity ligation assay in T-cell–APC conjugates. We found that in activated T cells, IRAP vesicles contain several components of the TCR signalosome, such as ZAP-70, LAT, Lck and CD3ε (Fig. 3f). Altogether these results demonstrate that the TCR duly signals from IRAP/Stx6^+^ compartments and that IRAP deletion impairs TCR signalling.

### IRAP S-acylation is important for TCR activation

**Fig. 4:**
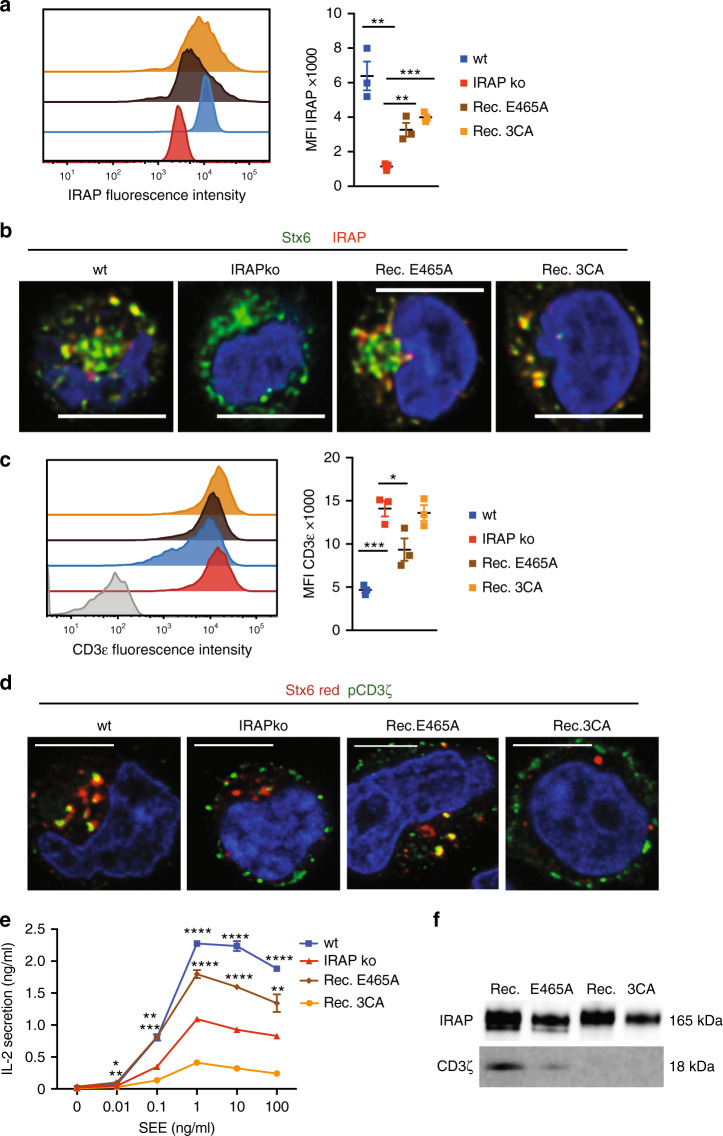
IRAP S-acylation is important for TCR activation. **a** Intracellular IRAP expression in wt, IRAP ko and IRAP ko cells reconstituted with IRAP E465A or IRAP 3CA, measured by flow cytometry (*n* = 3 experiments, **p_wt_ = 0.0034, **p_E465A_ = 0.0071, ***p = 0.0002). Values represent mean ± SEM of three independent experiments. **b** Representative images of Jurkat T cells expressing endogenous IRAP (wt) and IRAP ko cells expressing IRAP E465A or IRAP 3CA via lentiviral transductions. The cells were activated on CD3ε/CD28-coated slides for 10 min, fixed and stained with antibodies specific for IRAP and Stx6. Bars represent 5-μm scale. **c** CD3ε expression on the cell surface of wt, IRAP ko Jurkat T cells and IRAP ko reconstituted with IRAP E465A and IRAP 3CA, measured by flow cytometry (*n* = 3 experiments, ***p = 0.0006, *p = 0.0398). Values represent mean ± SEM of three independent experiments. **d** Representative images of Jurkat T cells expressing endogenous IRAP (wt) and IRAP ko cells expressing IRAP E465A or IRAP 3CA via lentiviral transductions. The cells were activated on CD3ε/CD28-coated slides for 10 min, fixed and stained with antibodies specific for IRAP and phospho-CD3ζ. Bars represent 5-μm scale. **e** wt, IRAP ko Jurkat T cells and IRAP ko transduced with lentiviruses expressing IRAP E465A or IRAP 3CA were incubated with SEE-pulsed Raji cells. Values represent mean ± SEM of three replicates of one representative experiment of three independent experiments. Wt: *p = 0.0116, **p = 0.0010, ****p < 0.0001, IRAP E465A: **p_0.01_ = 0.0037, ***p = 0.0001, ****p < 0.0001, **p_100_ = 0.0030. All *p*-values (**a**, **c**, **e**) were calculated with two-tailed unpaired *t* tests. **f** Immunoblot analysis of IRAP and CD3ζ after immunoprecipitation with anti-IRAP in IRAP ko Jurkat T cells reconstituted with IRAP E465A or IRAP 3CA. Images are representative of two independent experiments.

Since increasing evidence suggests that IRAP has a dual function, exerting apart from its aminopeptidase activity^13,21^ a role in the vesicular trafficking^14,22,23^, we initially wondered whether IRAP peptidase activity is directly required for T-cell activation. In addition, the cytosolic domain of the protein can be S-acylated, a modification likely affecting IRAP interaction with other proteins or/and IRAP localisation in lipid rafts^24^. This modification could be important in TCR trafficking and signalling, which has been shown to depend on other S-acylated proteins, such as flotillins^20^. To analyse the contributions of aminopeptidase activity and S-acylation of IRAP in T-cell activation, we reconstituted IRAP-deficient Jurkat cells with two forms of IRAP: a full-length protein lacking aminopeptidase activity due to a point mutation (IRAP E465A) in the active site^20^ (IRAP E465A) and a full-length protein with three cysteine residues (C35, C103 and C114) mutated to alanine (IRAP 3CA) hampering protein S-acylation^24^. Both forms of mutated IRAP were expressed at lower levels than the endogenous protein (Fig. 4a). As expected from previously published reports, IRAP E465A and IRAP 3CA showed the same intracellular localisation as the endogenous protein (Fig. 4b)^22,24^. However, only IRAP E465A partially restored the wild-type T-cell phenotype, as demonstrated by a decrease in TCR complex and in phosphorylated CD3ζ chain levels at the cell surface (Fig. 4c, d). In addition, only the IRAP E465A form was able to partially restore the IL-2 secretion after stimulation with SEE-loaded Raji B cells (Fig. 4e). These data demonstrate a requirement of IRAP S-acylation, but not enzymatic activity for proper TCR activation. Since S-acylation is known to facilitate protein–protein interactions, we immunoprecipitated IRAP E465A and IRAP 3CA proteins and analysed their interaction with CD3ζ by immunoblot. These experiments demonstrate that defective IRAP S-acylation abolishes IRAP–CD3ζ interactions (Fig. 4f).

### IRAP colocalizes with the TCR in primary murine T cells

**Fig. 5:**
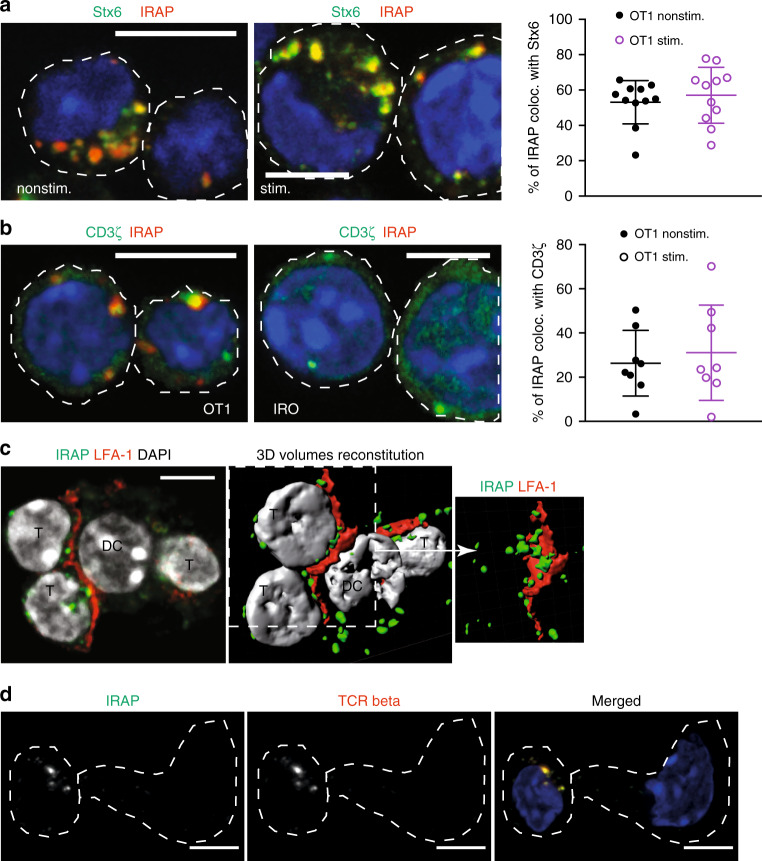
IRAP colocalizes with the TCR in primary murine T cells. **a** OT1 effector T cells were spread on poly-lysine (nonstim.) or CD3ε-coated (stim.) slides for 10 min, fixed and stained with antibodies specific for IRAP and Stx6. Each dot represents a cell (*n* = 11 cells per condition). Bars represent 5-μm scale. **b** OT1 T cells were spread on CD3ε-coated slides for 10 min, fixed and stained with antibodies specific for IRAP and the ζ chain (*n* = 8 cells per condition). Bars represent 5-μm scale. Images are representative of two independent experiments. **c** Confocal microscopy and tri-dimensional reconstitution of conjugates formed by SIINFEKL-pulsed dendritic cells and OT1 naive T cells. After fixation, the cells were stained with specific antibodies for LFA-1 (red), IRAP (green) and DAPI (grey). Images are representatives for two independent experiments. Bars represent 5-μm scale. **d** Confocal microscopy of conjugates formed by SIINFEKL-pulsed dendritic cells and OT1 naive T cells. After fixation, the cells were stained with specific antibodies for IRAP (green) and TCRb (red). Bars represent 5-μm scale. Images are representatives for two independent experiments.

To investigate the significance of the IRAP-dependent TCR signalling compartment in primary T cells, we crossed constitutive IRAP-deficient mice with Rag1-deficient, OT1 transgenic mice and named IRO the resulting IRAP^−/−^ Rag1^−/−^ OT1^+/+^ strain. In wt OT1 effector T cells, IRAP colocalized with Stx6 and CD3ζ, at the same levels as in Jurkat T cells (Fig. 5a, b; Supplementary Fig. 1a). We then formed conjugates between naive OT1 T cells and dendritic cells pulsed with the cognate peptide, SIINFEKL. We observed that in the conjugates IRAP endosomes were polarised toward the immune synapse (Fig. 5c) and contained the β chain of the TCR complex (Fig. 5d). Thus, in both naive and effector murine T cells IRAP colocalized with TCR components, not only in resting conditions but also after T-cell activation.

### IRAP-deficient mice have low numbers of peripheral T cells

**Fig. 6:**
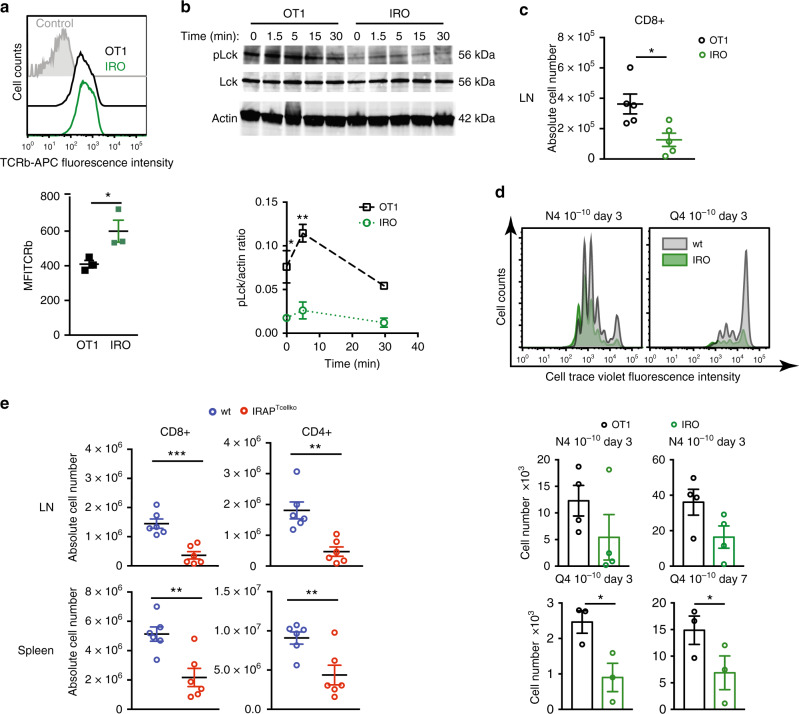
IRAP-deficient mice have low numbers of peripheral T cells. **a** TCRb expression on the cell surface of effector OT1 or IRO T cells (culture day 7), measured by flow cytometry. Each symbol represents an individual experiment. Values represent mean ± SEM of three independent experiments, *p = 0.0468. **b** Immunoblot analysis of effector OT1 or IRO T cells after activation for the indicated time points by anti-CD3ε/CD28 antibody. Values on quantification graphs represent mean ± SEM of pool of three independent experiments, *p = 0.0352, **p = 0.0031. **c** CD8^+^ T-cell absolute number quantification in OT1 or IRO mouse lymph nodes by flow cytometry. Values represent mean ± SEM of five mice from two independent experiments, *p = 0.0172. CD8^+^ cells are gated on CD45^+^TCRb^+^ live cells. **d** Quantification of number of OT1 and IRO T cells after activation by DC2.4 loaded with N4 or Q4 peptides. Values represent mean ± SEM of three or four independent experiments, Q4 day 3 *p = 0.017, Q4 day 7 *p = 0.011. Representative proliferation graphs at day 3 are shown. **e** CD8^+^ and CD4^+^ T-cell absolute number quantification in wt (IRAP^loxlox^) or IRAP^Tcellko^ mouse lymph nodes or spleen by flow cytometry. CD8^+^ and CD4^+^ cells are gated on CD45^+^TCRb^+^ live cells. Values represent mean ± SEM of six mice from two independent experiments, LN CD8 + ***p = 0.0003, CD4 + **p = 0.0017; spleen CD8 + **p = 0.0037, CD4 + **p = 0.0096. All *p*-values (**a**, **b**, **c**, **d**, **e**) were calculated with two-tailed unpaired *t* tests. For additional information, see Supplementary Figs. 4, 5, 6 and 7.

Considering that IRAP colocalized with the ζ and β chains of the TCR in primary mouse T cells, we wondered whether IRAP deletion affects TCR cell surface levels and TCR signalling. Similar to Jurkat T cells, when compared with OT1 T cells, IRO effector T cells showed increased TCR expression at the cell surface (Fig. 6a) and reduced Lck phosphorylation on the activating Tyrosine 394 (Fig. 6b). Surprisingly, IRO T cells divided equivalently to OT1 T cells upon CD3ε/CD28 activation (Supplementary Fig. 4a). However, when we analysed lymphoid organs of IRO mice, we observed a reduced number of cells in the lymph nodes (LN) (Fig. 6c). This suggested a defect either in the thymic selection or in the peripheral survival of the IRO T cells. The fact that both positive selection and peripheral survival are driven by low-affinity self-peptides^25^, raised the question of whether IRAP is of importance in primary T cells for suboptimal TCR activation. We then activated OT1 and IRO T cells by both, a high-affinity ligand (SIINFEKL, N4) and a lower-affinity ligand (SIIQFEKL, Q4)^26^ and monitored their divisions and survival for 7 days. Both OT1 and IRO T cells showed similar cell-division rates, but at low peptide concentrations IRO T numbers were decreased, presumably due to cell death (Fig. 6d). These results suggest that suboptimal TCR activation requires the presence of IRAP/Stx6^+^ intracellular signalling compartments.

To ascertain this hypothesis in the context of a polyclonal T-cell repertoire, we generated a mouse strain in which IRAP was deleted exclusively in T cells by crossing IRAP^loxlox^ mice^27^ with Lck-Cre mice^28^ and we called this mouse strain IRAP^Tcellko^. CD3ε^+^-sorted cells from IRAP^Tcellko^ spleens showed that IRAP was efficiently deleted in this strain (Supplementary Fig. 4b). IRAP^Tcellko^ mice displayed similar frequencies of naive, central memory and effector memory CD4^+^ and CD8^+^ T cells in peripheral lymphoid organs (Supplementary Fig. 4c, d), but a strong reduction in the absolute numbers of peripheral CD4^+^ and CD8^+^ T cells (Fig. 6e). This reduction, in agreement with the moderated lymphopenic phenotype already observed in IRO mice, was caused by IRAP deletion and not by Cre expression in T cells^29^ (Supplementary Fig. 5a, b).

As the IRAP compartment could intervene during T-cell selection in the thymus, we turned our attention to IRAP and Stx6 expression in early stages of T-cell development. ImmGen (http://rstats.immgen.org/Skyline_microarray/skyline.html) expression data show that IRAP expression is increased in T cells after the transition from double-negative (DN) to double-positive (DP) and single-positive (SP) thymic CD4^+^ and CD8^+^ T-cell populations in the thymus, having the highest expression in peripheral T cells^30^ (Supplementary Fig. 5c). Unexpectedly, Stx6 mRNA expression was not correlated with IRAP mRNA expression (Supplementary Fig. 5c). To measure IRAP and Stx6 expression at protein level, we performed an intracellular staining with specific antibodies. Flow cytometry analysis confirmed that IRAP expression progressively increases from DN to SP thymocytes, while Stx6 has the highest expression in thymocytes at the DP1 stage (Supplementary Fig. 5d). Based on these results, we expected that IRAP deletion would mostly affect the survival and function of mature T cells.

To investigate this hypothesis, we analysed the thymus of wt and IRAP^Tcellko^ mice. Analysis of IRAP^Tcellko^ thymus did not show a significant alteration of thymocyte populations (Supplementary Fig. 6a, b). The TCR and CD5 cell surface levels were equivalent between wt and IRAP-deficient thymocytes at all stages, while CD69 levels were slightly, but significantly, decreased in IRAP-deficient thymocytes in the DP2, DP3 and CD8 SP stages (Supplementary Fig. 6c). CD69 is an early activation marker, known to be rapidly upregulated after TCR stimulation and to disappear when the stimulation is withdrawn. In the thymus, it is transiently expressed by immature DP thymocytes that are undergoing positive or negative selection and its downregulation is important for final SP thymocyte emigration^31^. Since CD69 upregulation has been demonstrated to correlate with the strength of TCR activation^32^, the slightly decreased expression of CD69 on IRAP-deficient DP thymocytes might reflect a lower TCR signalling capacity in these cells. However, considering the fact that equal numbers of SP thymocytes are found in wt and IRAP^Tcellko^ thymi, the lower numbers of T cells in the periphery of IRAP^Tcellko^ mice could result from a lower survival capacity of IRAP^Tcellko^ T cells in the periphery. The tonic signal, which is triggered by low-affinity TCR interactions with self-pMHC in the periphery and is required for peripheral T-cell survival^33,34^ also appears to be affected by IRAP deletion, as suggested by constitutively low amounts of phosphorylated, active Lck in IRO cells (Fig. 6b). Thus, the intracellular IRAP/Stx6^+^ CD3ζ pool, which is diminished in the absence of IRAP, is likely essential for peripheral T-cell survival, probably through tonic signal triggering.

### IRAP is required for anti-tumour T-cell response

**Fig. 7:**
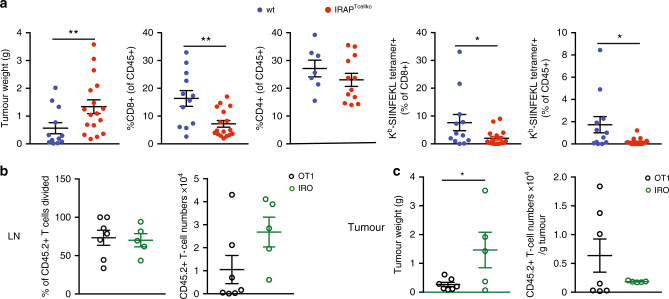
IRAP is required for anti-tumour T-cell response. **a** Wt or IRAP^Tcellko^ mice were injected s.c. with EG7-ova cells, and 12–14 days later, tumour weight and CD8^+^ T-cell response in the tumour were measured. Each dot represents a mouse. Values represent mean ± SEM of pooled results from three independent experiments. (Wt: *n* = 13 (except for graph, 3 where *n* = 7, IRAP^Tcellko^: *n* = 16 (except for graph 3, where *n* = 12). Graphs 1: **p = 0.0041, 2: **p = 0.0018, 4: *p = 0.0466, 5: *p = 0.0247). **b**, **c** CD45.1 C57BL6 mice were injected s.c. with EG7-ova cells, and 10–11 days later, 3 × 10^6^ CTV-labelled OT1 or IRO T were injected i.p. The divisions of transferred T cells and their absolute numbers were evaluated after 6 days in the lymph nodes (LN) (**b**). The tumour mass and the numbers of T cells infiltrating the tumour were analysed 6 days after T-cell transfer (**c**). Each dot represents a mouse. Values represent mean ± SEM of pooled results from two independent experiments (OT1: *n* = 7, IRO: *n* = 5, *p = 0.0440). All *p*-values were calculated with two-tailed unpaired *t* tests. For additional information, see Supplementary Fig. 8.

Despite the reduced numbers of peripheral T cells in IRAP-deficient mice, these cells were able to proliferate not only in vitro (Supplementary Fig. 4a) but also in vivo, as demonstrated by the equivalent numbers of ovalbumin-specific CD8^+^ T cells generated 14 days after immunisation with an AAV viral vector coding for ovalbumin^35^ (Supplementary Fig. 6d). However, considering the inability of IRAP-deficient T cells to efficiently respond to suboptimal TCR activation (Fig. 6d) and the importance of the first wave of low-affinity T cells recruited in pathogen-specific T-cell responses^26,36^, we wondered if IRAP-deficient T cells would control the priming of anti-tumour T-cell responses^25^. To investigate this, we injected subcutaneously IRAP^Tcellko^ and control mice with EL4-ovalbumin tumours and monitored tumour growth and tumour infiltration by T cells. Twelve days after tumour inoculation, IRAP^Tcellko^ mice showed a significantly increased tumour mass in comparison with the control mice, and a strongly reduced infiltration of the tumour not only by ovalbumin-specific CD8^+^ T cells but also by the whole CD8^+^ T-cell population (Fig. 7a; Supplementary Fig. 8a).

The low numbers of CD8^+^ T cells in the tumour could be a resulting consequence of low numbers of T cells in the periphery of IRAP^Tcellko^ mice or of a cell-intrinsic defect in TCR signalling in IRAP-deficient T cells. To discriminate between these two hypotheses, we performed an adoptive transfer experiment, in which the numbers of WT (OT1) and IRAP-deficient (IRO) antigen-specific T cells are normalised. Wild-type, CD45.1^+^ C57BL6 mice were inoculated subcutaneously with EL4-ovalbumin tumours. Ten days later, the mice having similar tumour mass were injected intraperitoneally with equal numbers of fluorescently labelled CD45.2^+^ OT1 transgenic cells or CD45.2^+^ IRO cells. At day 6 after their transfer, both OT1 and IRO cells displayed the same levels of cell divisions in the draining lymph nodes (Fig. 7b). Nevertheless, the tumour size for the mice that had received wt OT1 T cells was significantly lower and correlated with a higher number of infiltrating T cells (Fig. 7c; Supplementary Fig. 8b).

Therefore, even when the number of peripheral T cells was the same, the IRAP-deficient T cells failed to properly control the tumour growth. These results indicate that the intracellular pool of CD3ζ is required not only for T-cell survival in the periphery but also for the initiation of efficient T-cell responses against tumour cells.

## Discussion

It has been previously shown that the T cells have an abundant intracellular pool of CD3ζ chain. This pool is recruited to the IS by mechanisms that involve either independently, or in a synchronised manner, the VAMP7-synaptotagmin-7^3^ and Rab35-VAMP3 exocytic pathways^17,37^. Blocking these exocytic mechanisms prevents TCR enrichment at the IS, which suggests that the intracellular pool of the CD3ζ chain plays a crucial role in T-cell activation. Although the CD3ζ exocytic mechanisms are partially described, the identity and the origin of its intracellular pools remain unclear. In this work, we demonstrate that the SNARE Stx6^+^ endosomal compartment, whose stability depends on IRAP expression, hosts an intracellular pool of the CD3ζ TCR subunit. We show that upon TCR engagement, this intracellular pool of CD3ζ is crucial for stabilisation of the IS and for the amplification of TCR signalling, including signalling at the endosomal level, as previously proposed^18,19^. Endosomal signalling has been modelled to be much more efficient than plasma membrane signal diffusion^38^, and it offers the advantages of isolation of signalling molecules from the plasma membrane phosphatases, as well as the possibility of TCR interaction with signalling proteins residing in the cytosol.

We also demonstrate that this CD3ζ intracellular pool is formed via clathrin and DnM2-dependent endocytosis of CD3ζ from the plasma membrane. When this endocytosis was compromised, not only the CD3ζ chain but also IRAP were exclusively localised at the plasma membrane. From this observation and considering the interaction between IRAP and the ζ chain, we presume that the constitutive recycling of the ζ chain follows the slow constitutive recycling, characteristic for IRAP^13,39^. In this shared path, IRAP, which contains several endocytic motifs^13^, could serve as a guide to the ζ chain, which does not contain such motifs. Moreover, since IRAP has been recently shown to directly bind to the retromer^23^, its interaction with the ζ chain might avoid CD3ζ chain targeting to lysosomes and its degradation. IRAP could be thus the link between the TCR and the retromer that has already been demonstrated as essential for TCR recycling^40^. Altogether, these hypotheses indicate that the relationship between CD3ζ, IRAP and the retromer in TCR trafficking and signalling require further studies.

Our study confirmed that constitutive and activation-induced TCR endocytosis follow different routes. We show that IRAP deletion enhances TCR expression in basal conditions, presumably by a decrease in CD3ζ constitutive endocytosis. However, the activated TCR is similarly internalized by both wt and IRAP-deficient cells. This differential impact of IRAP deletion on TCR endocytosis might be explained by the use of distinct endocytic pathways by the resting and the activated TCR, with a prevalence of clathrin-mediated pathways for the resting TCR^7,17,41^ and clathrin-independent pathways for the activated one^42,43^. In both situations, the TCR can be recycled^44^, and the recycling of activated TCR consolidates the IS and might be essential in vivo, where repeated consecutive contacts between T cells and antigen presenting cells are frequently required for complete activation of T cells^45^. The recycling of the CD3ε chain can be directly assessed by flow cytometry using specific antibodies, while the recycling of the ζ chain is usually performed using reporter molecules, such as CD8-CD3ζ^46^. Using these methods, we show that the CD3ε subunit of the activated TCR is recycled, but not the CD8–CD3ζ reporter. However, this reporter only shows the recycling behaviour of the isolated ζ chain, because it cannot integrate the full TCR complex, and these results are probably more relevant for the CD3ζ-based chimeric antigen receptors (CAR) than for the TCR complex-associated endogenous ζ chain.

The recycling of the CD3ζ chain has been previously investigated by time-lapse videomicroscopy using YFP-tagged CD3ζ, which can integrate the TCR complex. This fusion protein is internalized from the IS in endosomes described by the small GTPase TC21 from which it can be recycled to the IS^8,47^. More recently, activated TCR recycling has been shown to use an endocytic network described by the membrane-organising proteins flotillins^20^, which are important for TCR recycling to the IS, but not for its endocytosis from the IS. Flotillins are highly S-acylated proteins that indirectly associate with the TCR within cholesterol-enriched membrane domains. Similar to flotillins, and other essential players of TCR signalling, such as Lck^48^ and LAT^49^, IRAP is also S-acylated^24^. Our results show that, unlike flotillins, IRAP is not required for the recycling of the activated CD3ε unit of the TCR. However, we find that IRAP S-acylation is essential for its interaction with the ζ chain of the TCR, for the IL-2 production after TCR triggering as well as for the regulation of CD3ε cell surface levels. Although further studies are required to establish the relationship between IRAP and flotillins, one possible scenario is that S-acylated IRAP in lipid rafts facilitates TCR retrieval by the retromer, as previously mentioned^23,24,40^.

Concerning the functional relevance of the IRAP-dependent intracellular pool of CD3ζ in primary T-cell activation, we demonstrate that it is particularly important for the outcome of suboptimal TCR–pMHC ligation, which is relevant for peripheral T-cell survival and for the initiation of effective T-cell responses against tumour cells.

Despite low numbers of peripheral T cells, we did not find major changes in thymocyte populations in the absence of IRAP, although we detected a slightly decreased expression of CD69 in IRAP-deficient DP thymocytes. This reduction in CD69 levels could be the consequence of weaker TCR signalling in IRAP-deficient DP thymocytes, but this signalling is sufficient to drive T-cell selection, as demonstrated by equal numbers of SP cells in wt and IRAP-deficient thymi. Thus, our results argue against a major role of IRAP in T-cell development that could be explained not only by the relatively low expression of IRAP in DN and DP cells, but also by the different behaviour of the thymocytic TCR. Thus, DP thymocytes make multifocal synapses with several centres of TCR surrounded by adhesion molecules and display a sustained tyrosine phosphorylation^50^. This sustained phosphorylation might be due to lower expression of inhibitory phosphatases in DP thymocytes^51^ than in mature T cells. Low phosphatase expression in thymocytes could also explain why DP thymocytes, despite their low TCR levels at the plasma membrane, are more sensitive to pMHC than mature T cells^1^.

In conclusion, we found that the constitution of a specific intracellular pool of CD3ζ, essential for TCR signalling in mature T cells, depends on IRAP. Our results also identify a new parameter for the optimisation of T-cell-based immunotherapies, for which CD3ζ signalling could be manipulated to sustain T-cell survival and T-cell activation with weak TCR–pMHC ligands. Finally, we believe that the characterisation of IRAP-dependent T-cell endosomal signalling platforms will pave the way for future investigations concerning the intracellular signalling of other major ITAM-coupled immune receptors, such as the Fc immunoglobulin receptors and the B-cell receptor.

## Methods

### Mice

IRAP^lox/lox^ mice^27^ from SY. Chai (Monash University, Australia) and Lck-cre mice^52^ from S. Lotersztajn (CRI, INSERM U1149, France) were crossed to obtain IRAP-lox^+/−^-Lck-cre^+^ progeny. Those mice were re-crossed with IRAP^lox/lox^ to obtain IRAP^lox/lox^Lck-cre^+^ (IRAP^Tcellko^) and IRAP^lox/lox^Lck-cre^−^ (wt) mice. For evaluation of the phenotype of the cre recombinase alone, IRAP^lox/lox^Lck-cre^+^ mice were crossed to C57Bl/6 mice obtained from Janvier. CD45.1 mice were obtained from Charles River Italy (stock: 494C57BL/6L Y5.1), and were crossed with C57BL/6JRj from Janvier Laboratories France, to obtain CD45.1.2. All mice were genotyped to verify presence or absence of Lck-cre, IRAP^lox/lox^ and off-targets (gKO) using specific primers (Supplementary Table 1). IRAP-deficient mice^53^ from S.Keller (Virginia University, USA) were crossed with Rag1-deficient, OT1 transgenic mice^54^ to obtain IRAP^−/−^ Rag1^−/−^ OT1^+/+^ mouse strain named IRO. Mice were bred in a specific pathogen-free (SPF) facility, with ambient temperature between 20 and 24 °C, humidity between 40 and 60% and uninterrupted light–dark cycle. Both male and females mice between 6 and 12 weeks of age were used for experiments. All animal experiments, including the tumour injections, were approved by the Comité d’éthique pour l’expérimentation animale Paris-Nord/N^o^ 121 (APAFIS #16488), additionally approved by the CRI U1149 Ethical Committee and by the French Committee for OGM (DUO n° 5643).

### Cells

Jurkat T cells were validated by SSTR method, and present 88% of homology with DSMZ Leibniz ACC 282. Jurkat T cells, EG7-ova^55^, Raji B cells (ATCC-CCL-86) and DC2.4 (Sigma-Aldrich SCC142) were cultured in IMDM supplemented with 10% FCS, 2 mM L-glutamine, 50 μM β-mercaptoethanol, 100 U/ml penicillin and 100 μg/ml streptomycin. Daju-A2^56^ and HEK293FT cells (Invitrogen, R70007) were cultured in DMEM supplemented with 10% FCS, 2 mM L-glutamine, 50 μM β-mercaptoethanol, 100 U/ml penicillin and 100 μg/ml streptomycin. In order to obtain OT1 effector T cells, 1 × 10^6^ naive OT1 (isolated from the spleen of OT1 mice) per well were cultured with 0.5 × 10^6^ irradiated DC2.4 loaded with 1 μg/ml N4 peptide in 24-well plates in IMDM supplemented with 10% FCS, 2 mM L-glutamine, 50 μM β-mercaptoethanol, 100 U/ml penicillin and 100 μg/ml streptomycin. Human IL-2 (0.04 U/μL, Peprotech) was added to the cells on days 3 and 6, and cells were diluted every 2–3 days to 1 × 10^6^ cells/ml. Cells were used on days 8–10.

### Plasmids

Rab4-GFP, Rab11-GFP, Rab27-GFP and Rab8-GFP were generous gifts from A. Alcover (Institut Pasteur, France)^3^. Lck-GFP and Lck(Y505F)-GFP^46^ were kindly donated by J. Rossy (University of New South Wales, Australia). pCDEF3-CD8-CD3ζ^57^ was a gift from A. Weiss (University of California, CA, USA). EGFP-N1-IRAP 3CA^24^ was a gift from L. Chamberlain (University of Strathclyde, UK). pHR-ZIP(WT) (#27134)^18^, lentiGuide-Puro (#52963) and lentiCas9-Blast (#52962)^58^ pCMV delta R8.2 (#12263) and pMD2.G (#12259) were purchased from Addgene.

### Cloning of Rab4b, CD8-CD3ζ and IRAP in a lentiviral construct

eGFP-Rab4b was cloned into pLVX-EF1α-IRES-puro, linearised with EcoRI, by recombination using specific primers (Supplementary Table 1). Recombination was done using the In-Fusion^®^ HD Cloning Kit (Takara Bio) according to the manufacturer’s instructions.

CD8–CD3ζ was amplified from pCDEF3-CD8–CD3ζ mouse IRAP wt and E465A were amplified from pIRES2-IRAPfull length-HA^20^, and mouse IRAP 3CA was amplified from EGFP-N1-IRAP 3CA with specific primers listed in the Supplementary Table 1. The purified PCR products were cloned into pHR-ZIP (WT) after plasmid digestion with PasI and MluI. Selected clones were verified by plasmid digestion and sequencing (Eurofins).

### Transfections

For expression of fluorescent fusion proteins, 1 × 10^6^ Jurkat T cells were electroporated with 10 μg plasmid in 100 μL Opti-MEM using the NEPA21 electroporator (300 V, 1 ms, Nepagene). Forty-eight to 72 h after transfection, expression was verified by flow cytometry, and the cells were used for immunofluorescence studies.

### Lentiviral shRNA knockdown or protein overexpression

The pLK0.1-puromycin plasmids coding for IRAP-specific shRNA (TRCN0000296804), DnM2-specific shRNA (TRCN0000006649) and a non-targeting shRNA (shNT) were purchased from Sigma-Aldrich. The MV5 plasmid (pNL-SIN-CMV-eGFP-AP2μ-shRNA) coding for AP2μ-specific shRNA was a kind gift from M. Schindler (University Hospital Tuebingen). The lentiviral particles were produced according to the protocol published by Tiscornia et al.^59^. Briefly, pLK0.1 plasmids encoding the shRNA were co-transfected with the packaging plasmids pCMVDelta8.2 and the envelope plasmid pMD2G into HEK-293-FT cells via calcium chloride transfection. Six hours post transfection, the buffer was exchanged for complete DMEM and virus-containing supernatant was collected 24 and 48 h post transfection. The viral supernatants were concentrated by ultracentrifugation. Jurkat T cells were seeded in 96-well round-bottom plates at 5 × 10^4^ cells per well, and transduced in the presence of 10 μg/ml polybrene. Following 90-min centrifugation at 37 °C and 950×*g*, the lentiviral mix was replaced with complete IMDM medium. The day after, puromycin was added to the cells at 5 μg/ml, and selected cells were used 4–5 days post transduction.

### CRISPR/Cas9 knockout of IRAP

Jurkat T cells were transduced with lentiCas9-Blast, selected with blasticidin and cloned. Selected clones were verified for the presence of Cas9 by immunoblot using a rat anti-FLAG antibody (L5, Biolegend). Cas9-positive clones were then transduced with lentiGuide-Puro (sgIRAP or sgNT) using the primers listed in Supplementary Table 1. Transduced cells were selected with puromycin and cloned. Clones with efficient knockout were verified by immunoblot.

### Retroviral overexpression of TCR-MelanA2

Retroviral particles were produced with the same protocol used for lentivirus, using pCL-Ampho (Bio-Techne, #NBP2-29541) as packaging plasmid and with only one round of retrovirus supernatant collection, 48 h after transfection. Jurkat T cells were transduced with freshly produced retrovirus pMSGV1-F5AfT2aB encoding for α and β chain of the F5 anti-MART1/A2 α/β TCR (TCR-MelanA2), in 24-well plates at 4 × 10^5^ cells per well in the presence of 6 μg/ml polybrene. Following 90-min centrifugation at 37 °C and 950×*g*, the cells were incubated at 37 °C, 5% CO_2_ overnight. The day after, the retroviral mix was replaced with complete IMDM medium, and transduction efficiency was evaluated 3 days after by flow cytometry with R-PE-labelled Pro5 MHC Pentamer A*02:01 ELAGIGILTV (ProImmune) staining. Pentamer-positive cells were sorted on a SONY SH800 sorter (Sony Biotechnology), and cultured further till obtaining the desired number for ELISA experiments.

### Quantitative RT-PCR

AP2μ-GFP + cells were first sorted on a BD FACSMelody Cell Sorter. The total RNA was extracted from 1 × 10^6^ Jurkat T cells with the Nucleospin RNA Plus kit (Macherey-Nagel) according to the manufacturer’s instructions. One microgram of the total RNA was reverse transcribed into cDNA with the iScript cDNA synthesis kit (Bio-rad). Quantitative PCR was performed with Luna Universal qPCR MasterMix (New England Biolabs) using the CFX-96™ Real-Time System PCR instrument from Bio-rad. The primers used to detect AP2μ and the housekeeping mRNAs are listed in Supplementary Table 1.

### IL-2 secretion measurement by ELISA

For activation by SEE: Raji B cells were resuspended at 10^6^ cells/ml, and 50 μl were seeded in 96-well flat-bottom plates. Jurkat wt or IRAP ko cells were resuspended at 10^6^ cells/ml, and 100 μl were added to the Raji B cells. In total, 50 μl SEE (Toxin Technology) was added at the indicated final concentrations, and supernatants were harvested after 6 h incubation at 37 °C, 5% CO_2_.

For activation by MART1 peptide: 15 × 10^3^ Daju-A2 cells per well were cultured in a 96-well flat-bottom plate overnight. The day after, MART1 peptide (ProImmune) was added at the indicated final concentrations, as well as 200 × 10^3^ Jurkat wt or IRAP ko cells expressing TCR-MelanA2. Supernatants were harvested after overnight incubation at 37 °C, 5% CO_2_.

IL-2 was measured by ELISA with the kit Human IL-2 DuoSet ELISA (R&D Systems) on a Tecan Infinite 200 microplate reader.

### Flow cytometry for mouse phenotyping at steady state

Cells were stained with Ghost Violet™ 510 Viability Dye (TONBO Biosciences) for dead cell exclusion, blocked with Fc block (2.4G2, BD Biosciences) and stained with the following anti-mouse antibodies from Biolegend: rat anti-CD4-BV785 (GK1.5), rat anti-CD44-PE-Cy7 (IM7), hamster anti-TCRb-APC (H57-597), rat anti-CD8-PerCP-Cy5.5 (53-6.7), rat anti-CD45-APC-Cy7 (30-F11), rat anti-CD25-FITC (3C7), rat anti-CD5-A700 (53-7.3), rat anti-CD127-BV421 (A7R34) and hamster anti-CD69-PE (H1.2F3) (for thymus samples only) or rat anti-CD62L-PE (MEL-14) (for spleen and lymph node samples only). All antibodies were diluted at 1/200, except for anti-CD4-BV785 (1/400) and anti-CD44-PE-Cy7 (1/700). AccuCheck counting beads (Thermo Fisher Scientific) were added to each sample in order to calculate absolute cell numbers. Samples were analysed on a Fortessa (BD Biosciences) instrument.

### Intracellular flow cytometry for IRAP and Stx6 expression

Cells isolated from mouse spleen and thymus were stained with Ghost Violet™ 510 viability dye (TONBO Biosciences) for dead cell exclusion, blocked with Fc block (2.4G2, BD Biosciences) and stained with the following anti-mouse antibodies from Biolegend: CD4-BV785 (GK1.5), CD44-PE-Cy7 (IM7), TCRb-APC (H57-597), CD8-PerCP-Cy5.5 (53-6.7) and CD45-APC-Cy7 (30-F11). Cells were then fixed and permeabilized using fixation and intracellular staining permeabilization wash buffer from Biolegend following the manufacturer’s instructions. Cells were stained with mouse anti-IRAP-AF594 (F5, Santa Cruz Biotechnology, dilution 1/20) or with rabbit a-Stx6 (ProteinTech, dilution 1/100) or monoclonal rabbit IgG (Cell Signaling Technology, dilution 1/500) followed by staining with a-rabbit AF594 (Thermo Fisher Scientific, dilution 1/200).

### In vivo injection with EG7-ova and analysis of ova-specific CD8 + T-cell response

Wt (IRAP^lox/lox^Lck-cre^−^) and IRAP^Tcellko^ (IRAP^lox/lox^Lck-cre + ) mice were injected s.c. with 2 × 10^6^ EG7-ova cells. Both male and female mice were used at 8 to 12 weeks of age. Tumour size was measured starting from day 7 when the tumour was visible and measurable. The greatest longitudinal diameter (L) and the greatest transverse diameter (w) were determined using an electronic digital Vernier calliper. Tumour volume was estimated by the modified ellipsoidal formula: Tumour volume = 1/2(L × w^2^)^60^. In addition to the tumour mass, an evaluation grid was established to monitor animal welfare each day. This included several criteria, such as weight loss, behaviour, posture and general appearance of the animal. Mice were euthanized by cervical dislocation on days 12–14, depending on the experiment, when tumours had grown well but were still under the limit point, defined as tumour mass >2500 mm^3^, weight loss or gain superior to 10% or modification of animal behaviour. Tumours were extracted, weighed and digested with collagenase D (Roche) and Dnase I (Thermo Fisher Scientific). After red blood cell lysis with RBC lysis buffer (Biolegend), cells were stained with Ghost Violet™ 510 Viability Dye (TONBO Biosciences) for dead cell exclusion and then with the R-PE labelled Pro5 MHC Pentamer H-2Kb SIINFEKL (ProImmune). Cells were then blocked with Fc block (2.4G2, BD Biosciences) to prevent non-specific binding and stained with the following rat anti-mouse antibodies: CD45-APC-Cy7 (30-F11), CD8-PerCP-Cy5.5 (53-6.7), CD4-BV785 (GK1.5), CD44-PE-Cy7 (IM7), I-A/I-E-biotin (M5/114.15.2), CD11b-FITC (M1/70), GR1-biotin (RB6-8C5), F4/80-biotin (BM8). All antibodies were from Biolegend and used at 1/200 dilution, except for anti-CD4-BV785 (1/400) and anti-CD44-PE-Cy7 (1/700). Samples were analysed on a Fortessa (BD Biosciences) instrument.

### T-cell adoptive transfer experiment

OT1 T cells were isolated from the lymph nodes of transgenic RAG2 ko-OT1 wt or IRO transgenic mice, resuspended in PBS and stained with CellTrace Violet dye (5 μM Thermo Fisher Scientific) for 20 min at 37 °C. 3 × 10^6^ CTV-labelled OT1 or IRO T cells were injected i.p. in CD45.1 or CD45.1.2-recipient mice, 10 days after EG7-ova tumour injection. Mice were euthanized either on day 14 or day 16. Cells from the lymph nodes, the spleen and the tumour were stained with mouse anti-mouse: CD45.1-APC-Cy7 (A-20), CD45.2-A700 (104), hamster anti-mouse TCRb-APC (H57-597), rat anti-mouse: CD8-PerCP-Cy5.5 (53-6.7), CD4-BV785 (GK1.5), CD44-PE-Cy7 (IM7), I-A/I-E-biotin (M5/114.15.2), CD11b-FITC (M1/70), GR1-biotin (RB6-8C5), F4/80-biotin (BM8) and 7-AAD for dead cell exclusion. All antibodies were from Biolegend, and were used at 1/200 dilution, except for anti-CD4-BV785 (1/400) and anti-CD44-PE-Cy7 (1/700). AccuCheck counting beads (Thermo Fisher Scientific) were added to each sample in order to calculate absolute cell numbers.

### CD3ε and CD3ζ endocytosis and recycling by flow cytometry

In total, 2 × 10^6^ CD8-CD3ζ wt or IRAP ko cells were stained with biotinylated mouse anti-human CD8α (OKT8, Thermo Fisher Scientific, diluted 1/100) or mouse anti-human CD3ε (OKT3, Biolegend, diluted 1/100) at 4 °C for 30 min. Both antibodies were biotinylated with the EZ-Link^®^ Sulfo-NHS-SS Biotinylation Kit (Thermo Fisher Scientific) following the manufacturer’s protocol. After washing the unbound antibodies, cells were incubated for 15 min at 37 °C to induce endocytosis. The remaining antibody on the cell surface was blocked with streptavidin-FITC (Biolegend, diluted 1/100) for 20 min at 4 °C. After washing the unbound streptavidin, cells were re-incubated at 37 °C for 15, 30 and 60 min. The increased MFI relative to time point 0 corresponds to the amount of recycled CD3ε or CD3ζ.

### Immunoprecipitations and Immunoblots

IRAP was immunoprecipitated with the rabbit anti-IRAP antibody^61^ kindly provided by Susanna Keller (Virginia University, USA) or with rabbit anti-IRAP (D7C5, Cell Signaling Technology) bound on Dynabeads™ Protein G (Invitrogen) following the manufacturer’s instructions. For detection by immunoblot, the following antibodies were used: rabbit anti-IRAP^61^ (provided by S. Keller, Virginia University), rabbit anti-IRAP (D7C5, Cell Signaling Technology), mouse anti-IRAP (3E1, Cell Signaling Technology), mouse anti-Lck (3A5, Santa Cruz Biotechnology), mouse anti-CD3ζ (6B.10.2, Santa Cruz Biotechnology), mouse anti-β-actin (AC-15, Sigma-Aldrich), rabbit anti-LAT (Millipore), mouse anti-CD247 (pY142) (K25-407.69, BD Pharmingen), rabbit anti-pLAT, rabbit anti-ZAP-70 (D1C10E), rabbit anti-pZAP70 (65E4), rabbit anti-pPLC*γ*1, rabbit anti-PLC*γ*1 and rabbit anti-pSrc (D49G4) (all from Cell Signaling Technology). All primary antibodies were used at 1/1000 dilution, except for anti-β-actin (1/10,000). All developing antibodies were goat anti-species coupled with HRP used at 1/20,000 dilution (Jackson ImmunoResearch). Cells were lysed in 50 mM Tris, 150 mM NaCl, 1% CHAPS supplemented with protease inhibitor complete (Roche) and phosphatase inhibitor cocktails 2 and 3 (Sigma-Aldrich). Lysate supernatants were resuspended in 1× Laemmli buffer and were separated by SDS-PAGE using Criterion 4–15% acrylamide gels (BioRad) in Tris-Glycine-SDS buffer. The proteins were transferred on PVDF membranes (BioRad) using a Trans-Blot^®^ Turbo™ Transfer System from BioRad. Membranes were blocked for 2 h in 4% non-fat milk and incubated overnight with each antibody, washed extensively and incubated for 5 min with Clarity™ Western ECL Substrate (BioRad). The chemiluminescence signal was acquired using a ChemiDoc™ Imaging System and the quantification was realised with the Image Lab software (BioRad).

### TCR signalling after activation with antibodies

Jurkat T cells were resuspended at 10^6^/ml, and 1 ml of cells was incubated with 125 ng/ml mouse anti-CD3ε (OKT3, Biolegend) and 250 ng/ml mouse anti-CD28 (CD28.2, Biolegend) for the indicated time (0, 1.5, 5, 15 and 30 min) in water bath at 37 °C. OT1 effector T cells were resuspended at 10^6^/ml, and 1 ml of cells was incubated with 1 μg/ml hamster anti-CD3ε (145-2C11, Biolegend) and 2 μg/ml anti-CD28 (37.51, Biolegend) for 15 min at 4 °C. Then, mouse anti-Armenian and Syrian hamster IgG1 were added for cross-linking at 0.75 μg/ml (G9456, BD Biosciences), and cells were transferred for the indicated time (0, 1.5, 5, 15 and 30 min) in water bath at 37 °C. At the end of each incubation, cells were immediately transferred to ice, and cold PBS was added to stop the activation. Cell lysates were analysed by immunoblot.

### Immunofluorescence microscopy

In total, 3 × 10^5^ Jurkat T cells were incubated for 10 min on slides coated with poly-L-Lysine (Sigma-Aldrich) either untreated or treated overnight at 4 °C with anti-CD3/CD28 (OKT3, CD28.2, Biolegend). For all experiments, the cells were fixed with 4% PFA prewarmed at 37 °C for 15 min and permeabilized with 0.2% saponin in PBS containing 0.2% BSA and stained in the same buffer. Primary antibodies used were: rabbit anti-calnexin (Sigma-Aldrich), mouse anti-CD3ε (OKT3, Santa Cruz Biotechnology), mouse anti-CD3ζ (6B.10.2, Santa Cruz Biotechnology), Alexa-Fluor 488 mouse anti-CD247 (pY142) (K25-407.69, BD Biosciences) rabbit anti-LAMP1 (Sigma-Aldrich), rabbit anti-Stx6 (ProteinTech), rabbit anti-IRAP (a generous gift from S. Keller, Virginia University, USA), Alexa-Fluor 594 rabbit anti-IRAP (F5, Santa Cruz), mouse anti-Lck (3A5, Santa Cruz Biotechnology), goat anti-EEA1 (Santa Cruz Biotechnology), rabbit anti-LAT (Millipore), rat anti-LFA-1 (BD Biosciences), rabbit anti-IRAP (D7C5), mouse anti-IRAP (3E1), rabbit anti-pZAP70 (65E4) and rabbit anti-pSrc (D49G4) (all from Cell Signaling Technology). All antibodies were used at 1/100 dilution, except mouse anti-CD3ζ (1/50), Alexa-Fluor 488 mouse anti-CD247 (pY142) (1/5) and Alexa-Fluor 594 rabbit anti-IRAP (1/20). Secondary antibodies coupled with Alexa fluorochromes from Molecular Probes (Thermo Fisher Scientific) and Alexa-Fluor 488 Alpaca anti-mouse IgG1 (Chromotek) were diluted at 1/100. Images were acquired on a Leica SP8 confocal microscope or, where specified, on an LSM 510 Zeiss. Image treatment, analysis and quantification were performed with ImageJ or Fiji software.

### Jurkat T-cell and Raji B-cell conjugates for microscopy

Raji B cells were resuspended at 2 × 10^6^ cells/ml in PBS and stained with CellTrace Violet Dye (5 μM Thermo Fisher Scientific) for 20 min at 37 °C. Labelling was stopped with the addition of full IMDM medium, cells were resuspended at 2 × 10^6^ cells/ml and incubated with wt or IRAP ko Jurkat T cells also at 2 × 10^6^ cells/ml for 30 min at 37 °C. Cells were resuspended at 6 × 10^6^ cells/ml in full IMDM medium with SEE (100 ng/ml, Toxin Technology) and incubated at RT for 30 min on poly-L-Lysine slides. Slides were washed with PBS before fixation. For live cell microscopy, CTV-labelled Raji B cells were incubated with SEE in Poly-L-Lysine IBItreat μ-channels (IBIDI) for 30 min. After washing, Jurkat Cas9 gNT or Cas9 IRAP ko cells transduced with pHR-ZIP were added, and IS formation was followed on an LSM 510 Zeiss.

### TIRF microscopy

Poly-L-Lysine IBItreat μ-channels (IBIDI) were left untreated or coated overnight at 4 °C with anti-CD3/CD28 (OKT3, CD28.2, Biolegend). IN total, 3 × 10^5^ Jurkat T cells were incubated for 10 min at 37 °C, fixed, permeabilized, stained and left in PBS. Before imaging cells, TIRFM angle was set up to provide an evanescent field of thickness of around 100 nm. Cells were manually segmented to obtain regions of interest (ROI), and their areas were measured. Then, within each ROI, microclusters in the evanescent field were defined as signal intensity maxima detected by using the “Find Maxima…” function, for which a value of noise tolerance was arbitrarily set according to background for each experiment. Using this method allowed the discrimination of maximas coming from clusters (local bright patches at the plasma membrane or just below in the limit of thickness of the evanescent field) from a homogeneous signal. The number of “maximas” was then counted for each ROI, giving a cell-by-cell quantification of the number of microclusters at or below the plasma membrane. Images were acquired on a TIRF 3 Zeiss AxioObserver with objective 100×, ON1.4 with an Evolve EMCCD camera.

### FLIM microscopy

pHR-ZIP Jurkat T cells transduced with shNT or shIRAP or Jurkat Cas9 gNT or Cas9 IRAP ko cells transduced with pHR-ZIP were incubated for 10 min on slides coated with poly-L-Lysine (Sigma-Aldrich) and treated overnight at 4 °C with anti-CD3/CD28 (OKT3, CD28.2, Biolegend). After fixation and permeabilization, cells were stained with rabbit anti-IRAP followed by a secondary anti-rabbit Alexa405 (Thermo Fisher Scientific). Images were acquired on Leica TCS SP8 SMD (Single Molecule Detection), and GFP average lifetime was measured on a Leica TCS SP8 SMD system equipped with the FRET analysis tool and the SymPhoTime Software.

### Image analysis

Marker colocalizations were evaluated using only non-saturated images and the ImageJ software. A manual threshold was established for each channel before image analysis. Individual cells were delimitated with the freehand selection tool, and considered as region of interest (ROI) in ImageJ. For colocalization studies, all images were first translated to a binary image (black pixel intensity = 0; white pixel intensity = 1). The binary images for independent channels were multiplied to create a mask that encompasses the pixels present in both channels. The areas of pixels for each colour and of mask pixels were calculated using the plugin “measure stack” of ImageJ. The percentage of pixels for one colour that colocalized with pixels for another colour was calculated as the ratio: sum of area of pixels in the mask divided by sum of area of pixels from the first colour. Statistical analysis was performed with GraphPad Prism software using unpaired *t* tests. Tri-dimensional reconstitution of images was realised with Imaris Software.

### Proximity ligation assay (Duolink)

Duolink™ (OLINK Bioscience) was performed according to the manufacturer’s instructions. Briefly, the cells were incubated for 30 min on IBItreat μ-channels (IBIDI) coated with poly-L-Lysine, fixed for 10 min in 4% paraformaldehyde prewarmed at 37 °C, permeabilized in PBS, 0.2% saponin for 10 min and blocked with 0.2% BSA in PBS, 0.1% saponin. Primary antibodies used were: mouse anti-CD3ε (OKT3, Biolegend), mouse anti-CD3ζ (6B.10.2, Santa Cruz Biotechnology), rabbit anti-IRAP (a generous gift from S. Keller, Virginia University, USA), mouse anti-Lck (3A5, Santa Cruz Biotechnology), rabbit anti-LAT (Millipore), rabbit anti-Rab7 (H-50, Santa Cruz Biotechnology) and rabbit anti-IRAP (D7C5), mouse anti-IRAP (3E1) and rabbit anti-ZAP-70 (D1C10E) (all from Cell Signaling Technology). All primary antibodies were diluted at 1/100, except mouse anti-CD3ζ (1/50). After washing the cells, PLA probes were added, followed by hybridisation, ligation, and amplification for 100 min at 37 °C. Protein interactions were visualised after incubation with the detection solution. Fluorescence signal was acquired on an LSM 510 Zeiss confocal microscope.

### Transgenic OT1 T-cell isolation and proliferation assay

OT1 T cells were isolated from the lymph nodes of transgenic RAG2 ko-OT1 wt or IRO transgenic mice, resuspended in PBS and stained with CellTrace Violet Dye (5 *μ*M Thermo Fisher Scientific) for 20 min at 37 °C. DC2.4 cells were loaded with N4 (SIINFEKL) or Q4 (SIIQFEKL) at 10^−6^, 10^−8^ and 10^−10^ μM in the presence of 1 μg/ml LPS for 1 h at 37 °C. The cells were then irradiated and plated on 96-well flat-bottom plates at 25 × 10^3^ cells/well, and OT1 T cells were added at 50 × 10^3^ cells/well. T-cell proliferation was followed starting after 2 days of incubation by measuring CellTrace Violet dye dilution by flow cytometry after exclusion of dead cells by 7-AAD (Biolegend) and selection of T cells by TCRb staining using a Fortessa (BD Biosciences) instrument.

### Recombinant AAV vector production and immunisation

cOVA-expressing construct and recombinant AAV2/1-pseudotyped vectors were prepared as previously described^35^. Briefly, the cOVA cDNA was inserted in a pSMD2 AAV2 plasmid between the hPGK promoter and a polyA signal to create the pSMD2-cOVA construct. rAAV1-pseudotyped vectors were then prepared by co-transfection, in 293 cells, of the pSMD2-cOVA plasmid with the pXX6 and pAAV9pITRCO2 plasmids, encoding, respectively, for the adenovirus helper functions and the rep and cap genes. Vector particles were purified on iodixanol gradients from cell lysates obtained 48 h after transfection, and titers were measured by quantitative real-time PCR. For intramuscular immunisation, mice were anaesthetised and 10^10^ vg of rAAV2/1-cOVA, diluted in a final volume of 25 μL of 1× phosphate-buffered saline (1X PBS), was injected into the tibialis anterior using a 30-G RN Hamilton syringe. Ovalbumin-specific CD8 + T cells were detected in the blood on day 14 using K^b^-SIINFEKL pentamers.

### Statistical analysis

Statistical analysis was performed with GraphPad Prism software using unpaired two-tailed Student’s *t* test.

### Reporting summary

Further information on research design is available in the Nature Research Reporting Summary linked to this article.

## Supplementary information

Supplementary Information

Reporting Summary

Description of Additional Supplementary Files

Supplementary Movie 1

Supplementary Movie 2

Supplementary Movie 3

Supplementary Movie 4

## Data Availability

Full scans of the gels and blots are available in Supplementary Fig. 9 and in the Source Data file. Raw data for all figures and supplementary figures are also available in the Source Data file. All other data are included in the supplemental information or available from the authors upon reasonable requests.

## Source Data

Source Data
