# Supplementary material for: IRAP-dependent endosomal T cell receptor signalling is essential for T cell responses
Source: Nat Commun. 2020 Jun 2;11:2779. doi: 10.1038/s41467-020-16471-7 (PMC7265453; doi:10.1038/s41467-020-16471-7)
Supplement: Supplementary file 1 — Supplementary Information [file 41467_2020_16471_MOESM1_ESM.pdf]

Supplementary Information PDF

IRAP-dependent endosomal T cell receptor signalling is essential for T cell responses

Evnouchidou *et al.*

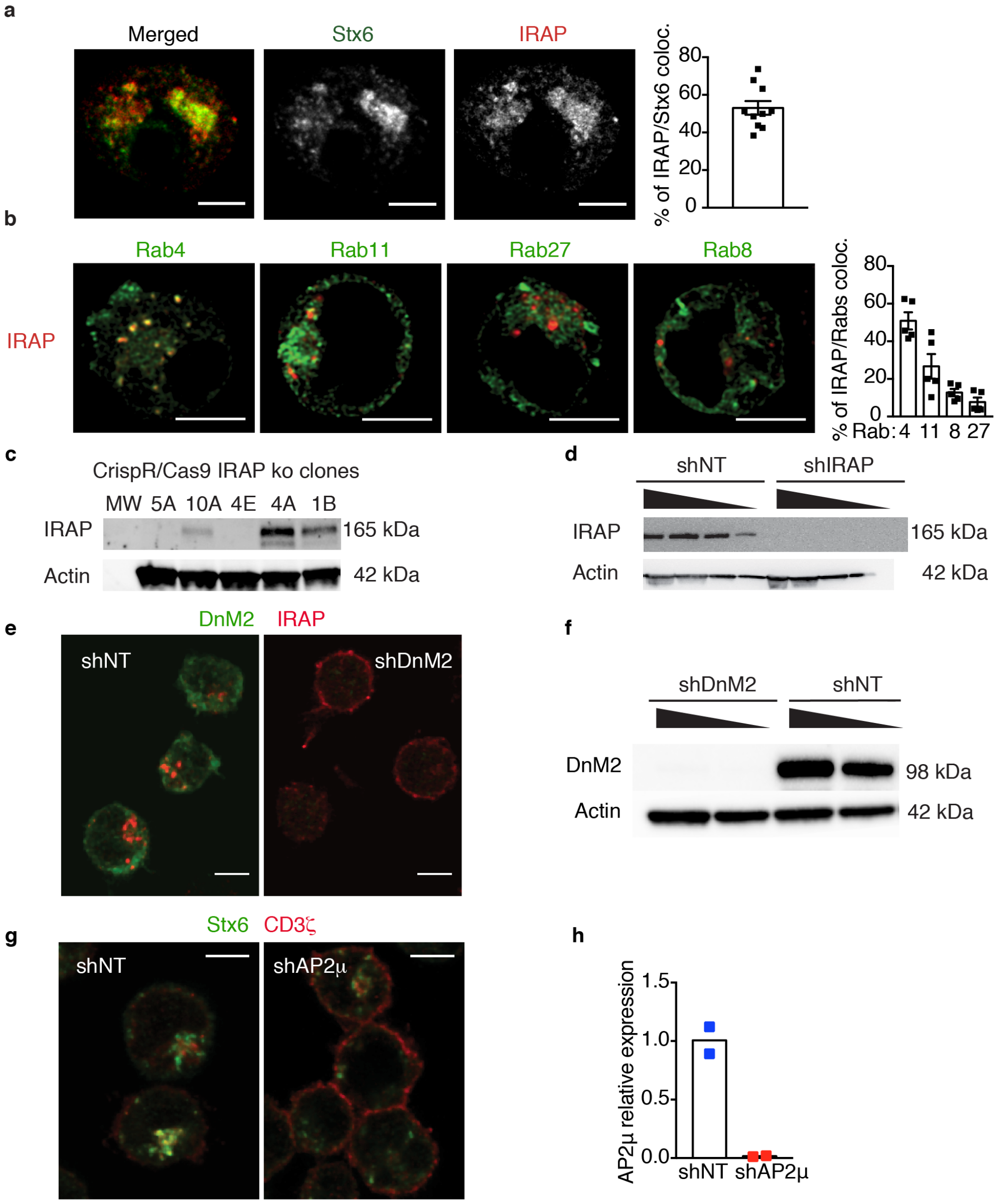

Supplementary Fig. 1. The CD3ζ intracellular pool colocalizes with IRAP and Stx6. (**a-b**, **e-g**) Confocal microscopy of Jurkat T cells. Bars represent 5 µM scale. Each dot represents an individual cell from 2 independent experiments. Values represent mean ± SEM. (**a**) Colocalization of Stx6 (green) with IRAP (red) and quantification (n=10). (**b**) Colocalization of IRAP (red) with Rab4, Rab11, Rab27, Rab8 (green) and quantification (for each condition, n=5). (**c**) Immunoblot analysis of CrispR/Cas9 IRAP ko clones. Clones 5A and 4E were selected to be used further. The screening was performed once. (**d**) Immunoblot analysis of IRAP in Jurkat T cells transduced with non-targeting (shNT) and anti-IRAP (shIRAP) shRNA. The image is representative for 5 individual experiments. (**e**) Colocalization of IRAP (red) with DnM2 (green) in wt and DnM2-depleted Jurkat T cells. The pictures are representative for 2 independent experiments. (**f**) Immunoblot analysis of DnM2 in Jurkat T cells transduced with non-targeting (shNT) and anti-DnM2 (shDnM2) shRNA. The image is representative for 3 independent experiments. (**g**) Colocalization of CD3ζ with Stx6 in wt or AP2µ-depleted Jurkat T cells. The pictures are representative for 2 independent experiments. (**h**) Quantification of RT-qPCR analysis of AP2µ relative expression in wt or AP2µ-depleted Jurkat T cells. Each dot represents an individual experiment, n=2.

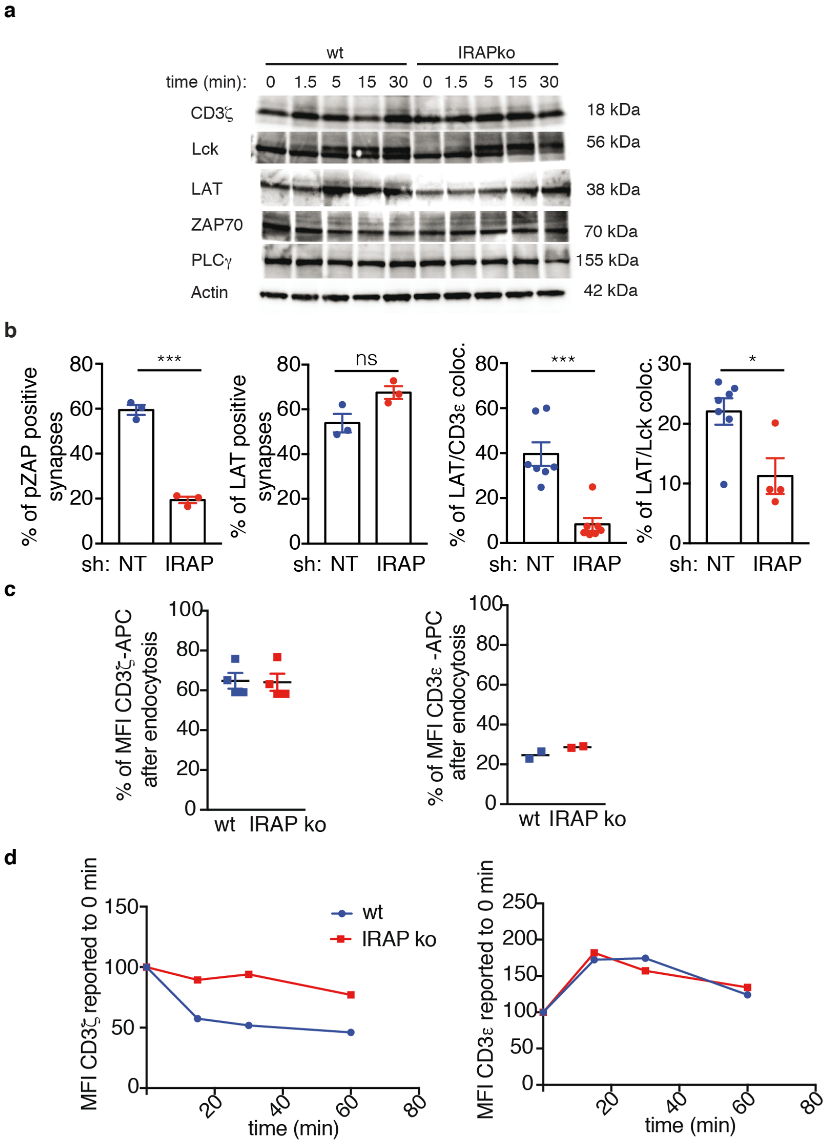

Supplementary Fig. 2. IRAP and the CD3ζ intracellular pool are required for proper TCR signalling and activation of Jurkat T cells. (**a**) Immunoblot analysis of total protein expression in wt or IRAP ko Jurkat T cells after activation by CD3ε/CD28 antibodies for the indicated times. Images are representative for 3 individual experiments. (**b**) Quantification of pZAP-70 and LAT positive synapses and of LAT/CD3ε and LAT/Lck colocalization at the synapse level. Lck, pZAP-70 and CD3ε are recruited much less to the synapse level in shIRAP cells. Each dot represents an individual experiment for pZAP (n=3, ***p=0.0001) and LAT (n=3) positive synapses and an individual cell for LAT/CD3ε (n=7, ***p=0.0002) and LAT/Lck (n=7 wt and n=4 ko, *p=0.016) colocalizations. Bars represent mean ± SEM. (unpaired, two-sided Student's *t*-test). Images are representative for 2 individual experiments. (**c**) Quantification of remaining expression on the cell surface after 15 min endocytosis measured by flow cytometry. Values represent mean ± SEM of 4 independent experiments for CD3ζ and 2 independent experiments for CD3ε. (**d**) Recycling of CD3ζ and CD3ε expressed as MFI increase relative to time point zero. Values represent mean of 2 independent experiments.

a

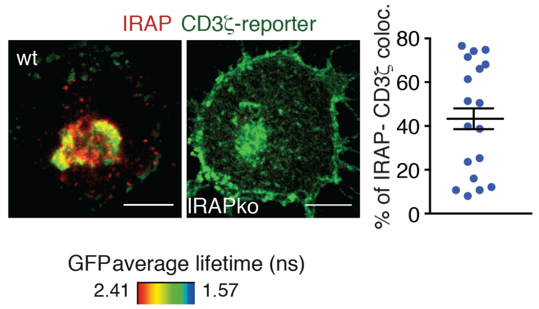

b

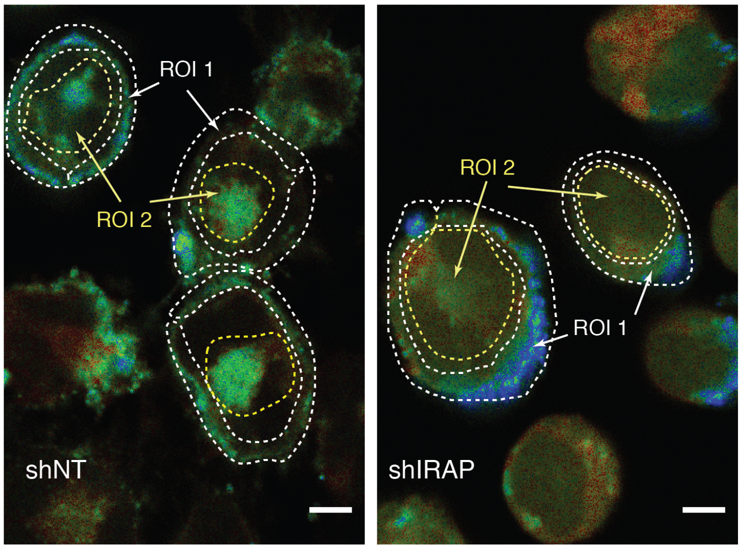

Supplementary Fig. 3. The CD3ζ reporter shows similar cell distribution and colocalization with IRAP as the endogenous CD3ζ. (**a**) Colocalization of the CD3ζ reporter with IRAP in wt versus IRAP ko Jurkat T cells. Bars represent 5 µM scale. Each symbol represents an individual cell (n=21). Values represent mean ± SEM. (**b**) Quantification of plasma membrane and endosomal GFP lifetime was realised with SymPhoTime Software, using for each cell two regions of interest (ROI), as depicted: ROI1 was considered as plasma membrane associated FRET-FLIM signal, while ROI2 as endosomal FRET-FLIM signal. Bars represent 5µM scale. All images are representative for 3 individual experiments.

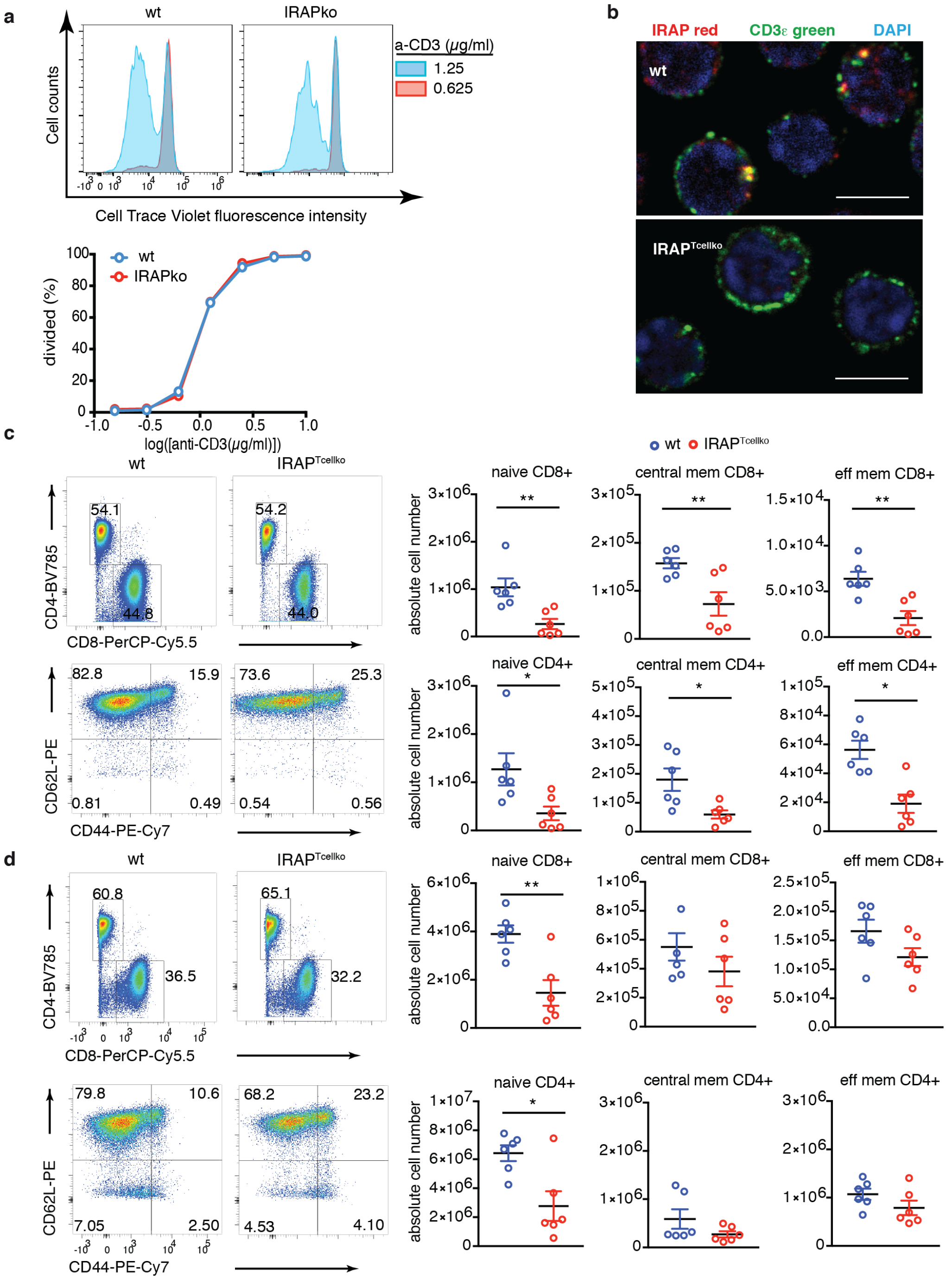

Supplementary Fig. 4. IRAP ko T cells divide as well as wt cells after strong activation and IRAP^{Tcellko} mice display similar frequencies of T cells in peripheral lymphoid organs but a significant lymphopenia. (**a**) Representative proliferation graphs for wt and IRAP ko mouse T cells after activation by CD3ε/CD28. Quantification graph is representative of 3 independent experiments. (**b**) Confocal microscopy analysis of IRAP (red) and CD3ε (green) in CD3$^+$ sorted cells from IRAP^{Tcellko} versus wt mice. Bars represent 5 µM scale. (**c-d**) Flow cytometry analysis of IRAP^{Tcellko} (**c**) lymph node (naïve CD8+ **p=0.0051; central mem. CD8+ **p=0.0096; eff.mem.CD8+ **p=0.0026; naïve CD4+ *p=0.0300; central mem. CD4+ *p= 0.0155; eff. mem. CD4+ **p=0.0020) and (**d**) spleen phenotype (naïve CD8+ **p=0.0035; naïve CD4+ *p=0.0104). CD8$^+$ and CD4$^+$ cells are gated on CD45$^+$TCRb$^+$ live cells and CD62L/CD44 graph shown is gated on CD45$^+$TCRb$^+$CD8$^+$ live cells. Values represent mean ± SEM of 6 mice from two independent experiments. All p values (**c**, **d**) are calculated with unpaired two-sided Student's *t*-test.

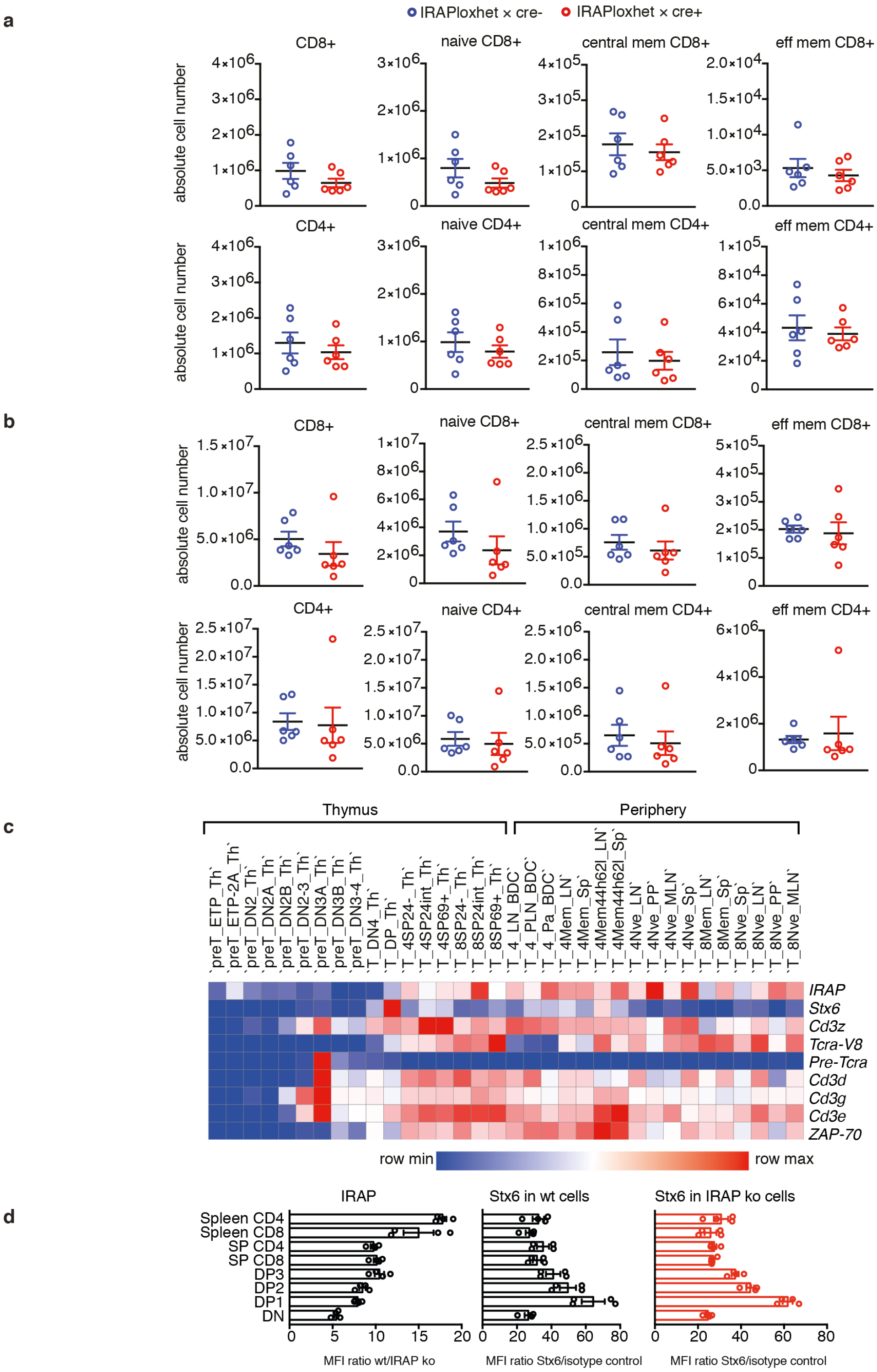

Supplementary Fig. 5. Phenotype of IRAPloxhet×cre mice and IRAP expression in thymus and mature T cells (**a-b**) Absolute cell number quantification in mice by flow cytometry. Values represent mean ± SEM of 6 mice from two independent experiments. $CD8^+$ and $CD4^+$ cells are gated on $CD45^+TCRb^+$ live cells: (**a**) IRAPloxhet×cre lymph node phenotype (**b**) IRAPloxhet×cre spleen phenotype. (**c**) Immgen profile of *IRAP*, *Stx6, ZAP-70* and TCR complex chain (*Tcra-V8, Pre-Tcra, Cd3d, Cd3e, Cd3g*) expression in various T cell populations. (**d**) IRAP and Stx6 expression in mouse spleen and thymus T cell subpopulations by flow cytometry. Each dot represents a mouse. Values represent mean ± SEM of 4 mice from 2 independent experiments.

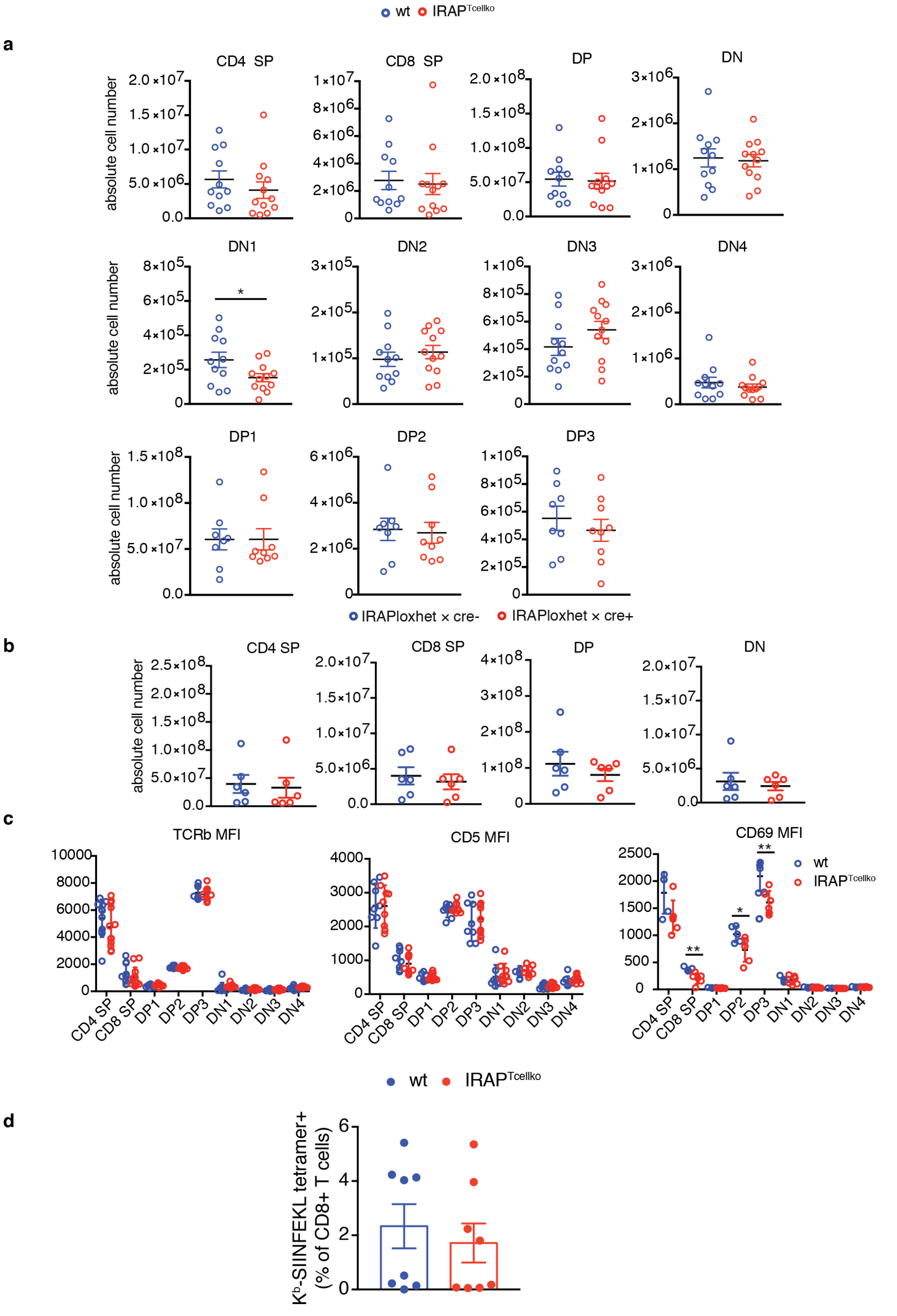

Supplementary Fig. 6. T-cell specific deletion of IRAP effect on thymus and the T cell response in IRAP^{Tcellko} mice after AAV-ova vaccination. (**a-b**) Absolute cell number quantification in mice by flow cytometry. CD8^{+}/CD4^{+} graph is gated on CD45^{+} live cells: (**a**) IRAP^{Tcellko} thymus phenotype (n=11 wt and 12 IRAP^{Tcellko} mice from three independent experiments except for DP1, DP2 and DP3 where n=8 wt and 9 IRAP^{Tcellko} mice from two independent experiments). (**b**) IRAP$^{loxhet×cre}$ thymus phenotype (DN1 *p=0.0490). (**c**) Comparison of MFI for TCRb, CD5 and CD69 in thymus T cell subpopulations. Each dot represents a mouse from 2 independent experiments (left panel n as in (**a**); middle panel n=8 wt and 9 IRAP^{Tcellko} mice; right panel n=5 wt and 6 IRAP^{Tcellko} mice). (CD69 MFI CD8+ SP **p=0.0055; DP2 *p=0.0350; DP3 **p=0.0096). (**d**) Ovalbumin-specific CD8^{+} T cell response in IRAP^{Tcellko} mice after vaccination with AAV-ova. Each dot represents a mouse from 2 independent experiments. Values represent mean ± SEM. All p values (**a**, **c**) are calculated with unpaired two-sided Student's *t*-test.

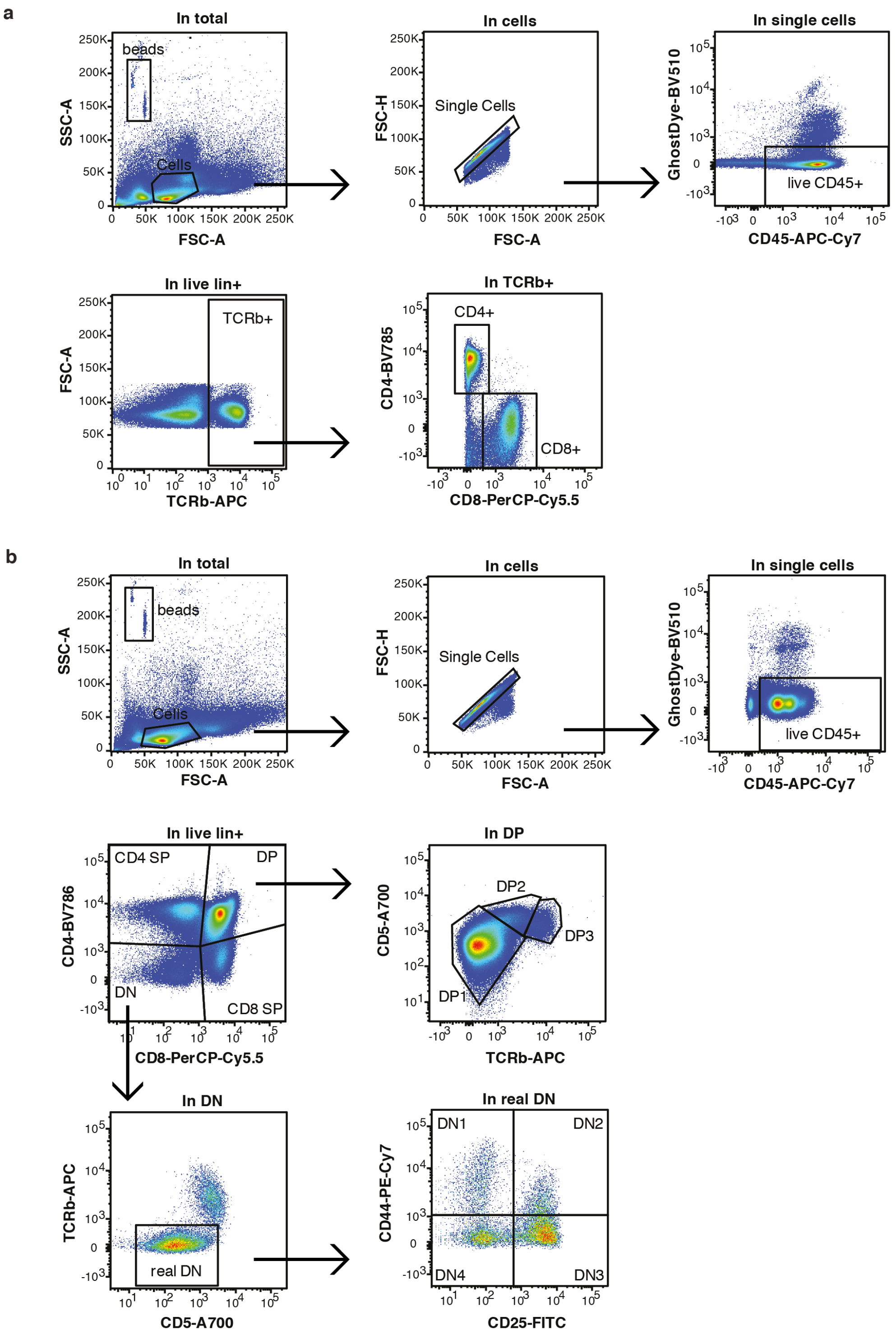

Supplementary Fig. 7. Representative gating strategy for T cell subpopulations at steady state in lymph nodes, spleen and thymus. (**a**) Representative gating strategy for lymph nodes and spleen for data presented on Fig. 6c, e, Sup. Fig.4c, d and Sup. Fig. 5a, b, d. Cells were pregated on leucocytes by FSC-A vs SSC-A and doublets were excluded by FSC-A vs FSC-H. TCRb+ cells were gated on live CD45+ cells. Within the TCRb+ gate, cells were further classified as CD4+ or CD8+. (**b**) Representative gating strategy for thymus for data presented on Sup. Fig. 6a,b,c. After gating on live CD45+ cells, cells were classified as DN, DP, CD4 SP and CD8 SP. The DP subset was further divided in DP1, DP2 and DP3 cells. The DN population was more precisely gated as negative for TCRb (real DN) and was further divided in DN1, DN2, DN3 and DN4 cells.

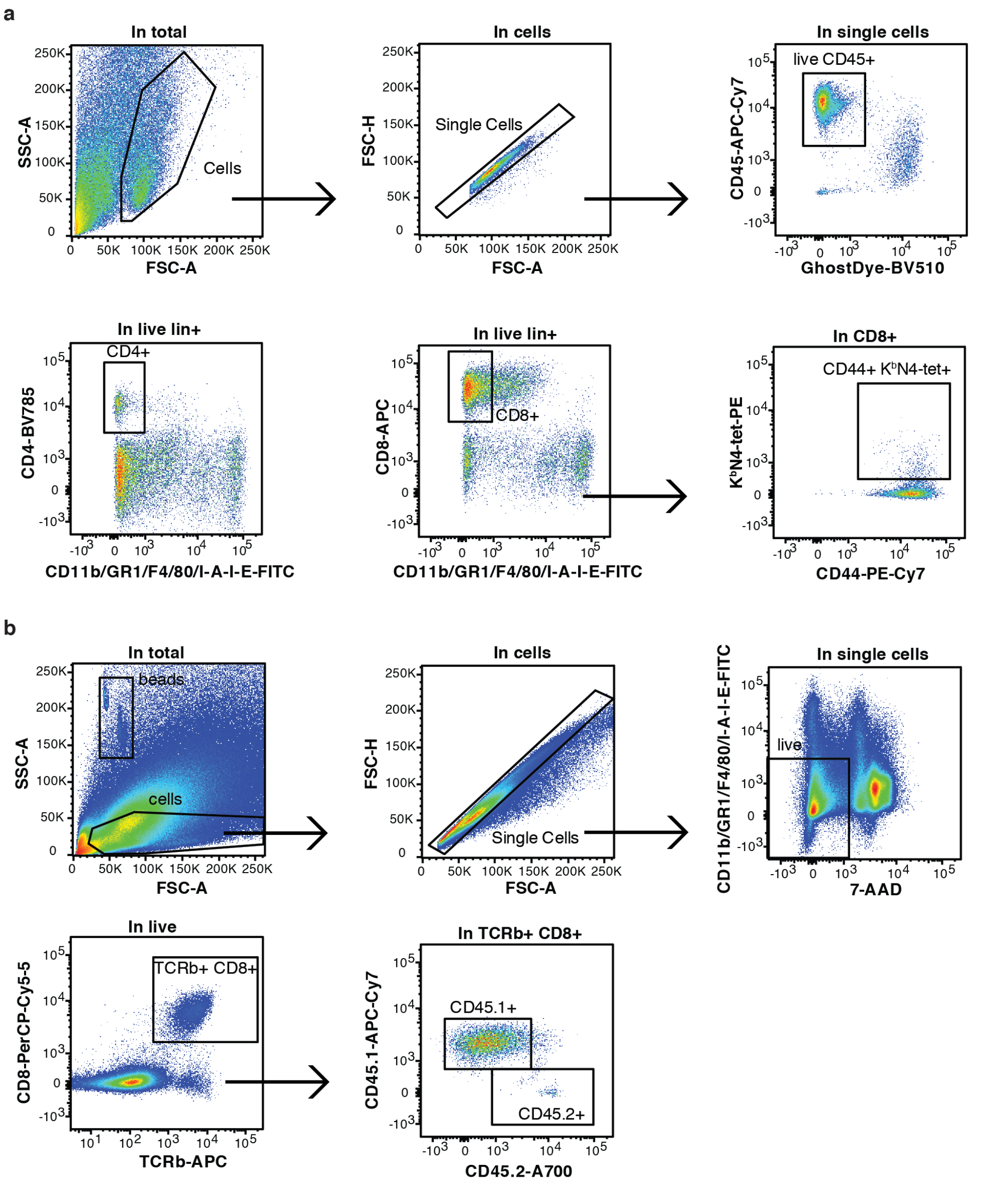

Supplementary Fig. 8. Representative gating strategy for T cell subpopulations in EG7-ova tumour. (a) Representative gating strategy for ova-tetramer detection in tumour in wt or IRAP^{Tcellko} mice presented on Fig. 7a or blood presented on Sup. Fig. 6d. Cells were pregated on leucocytes by FSC-A vs SSC-A and doublets were excluded by FSC-A vs FSC-H. Within the live CD45+ cells, cells were further classified as CD4+ or CD8+ after exclusion of (CD11b, GR-1, F4/80 and I-A/I-E)+ cells. CD44+-K^{b}N4-tetramer+ cells were gated inside the CD8+ population. (**b**) Representative gating strategy for adoptive transfer experiment presented on Fig. 7b. TCRb+CD8+ cells were gated on live-(CD11b, GR-1, F4/80 and I-A/I-E)- cells. OT1 or IRO injected cells were recognized as CD45.2+ whereas host cells were CD45.1+ (or CD45.1.2+ in some cases).

Supplementary Figure 9 Uncropped Original Scans

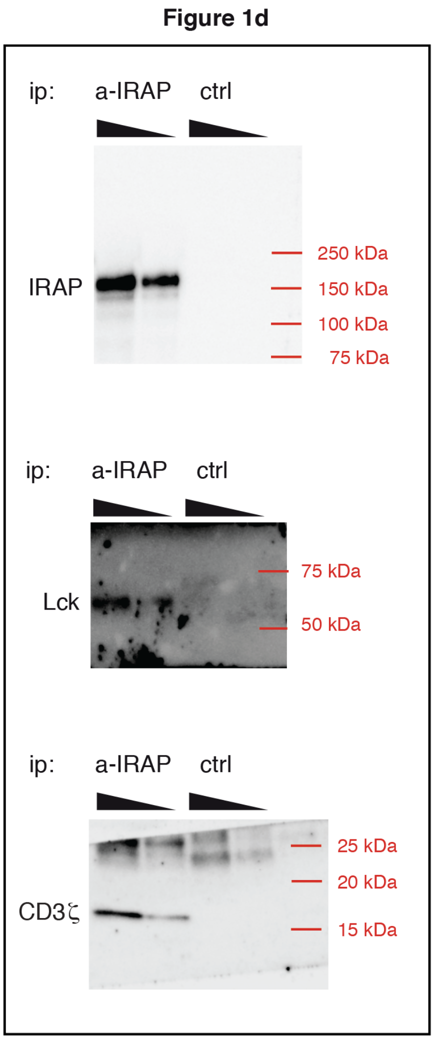

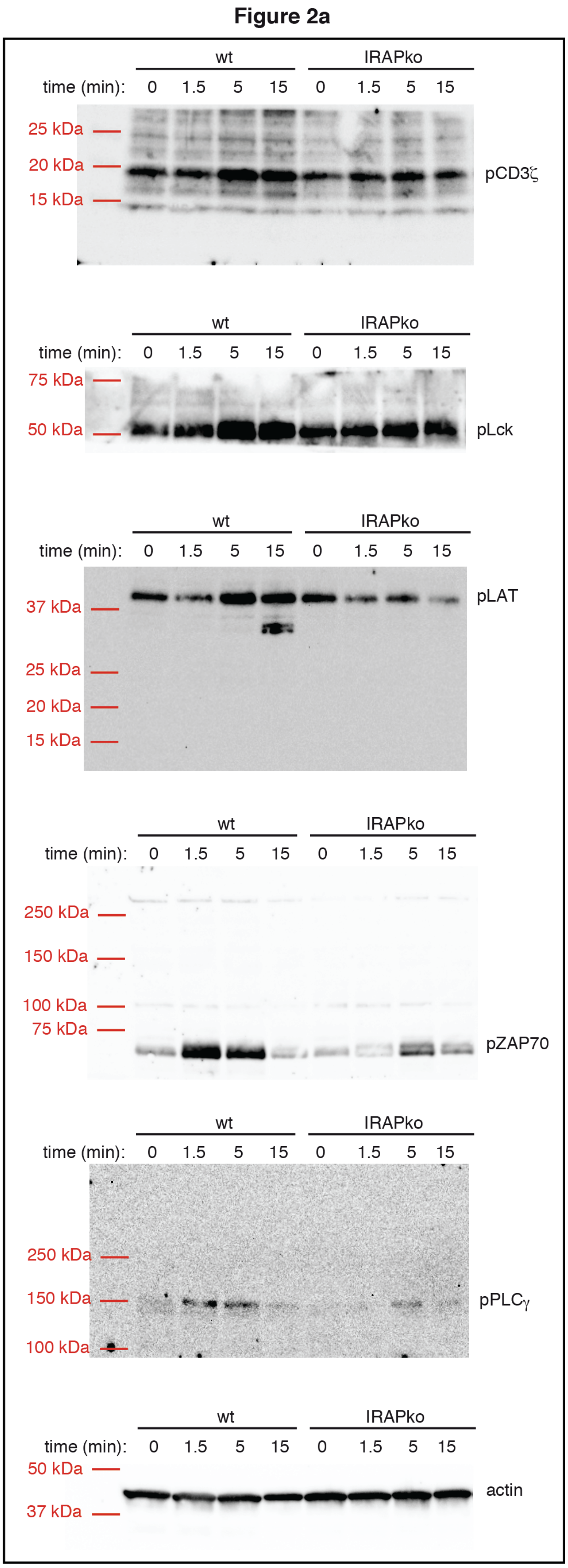

Supplementary Figure 9 Uncropped Original Scans

Figure 4f

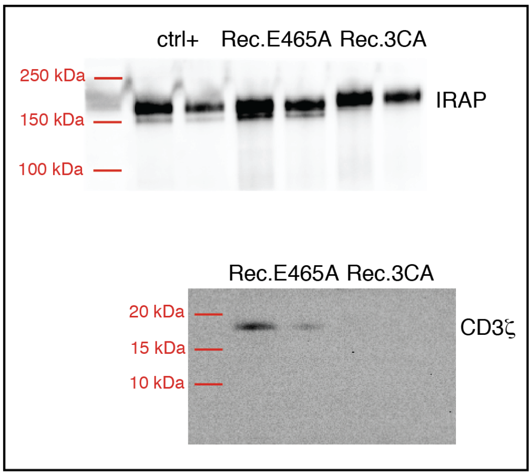

Figure 6b

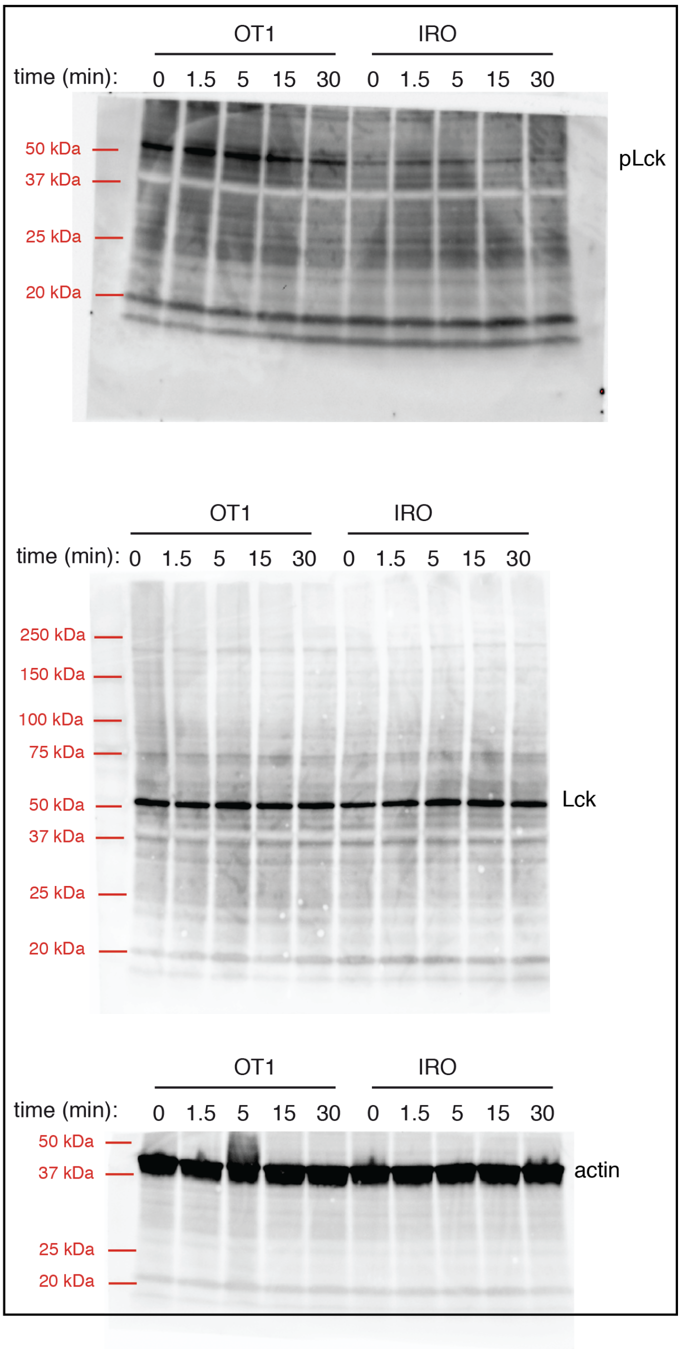

Supplementary Figure 9 Uncropped Original Scans

Supplementary Figure 1c

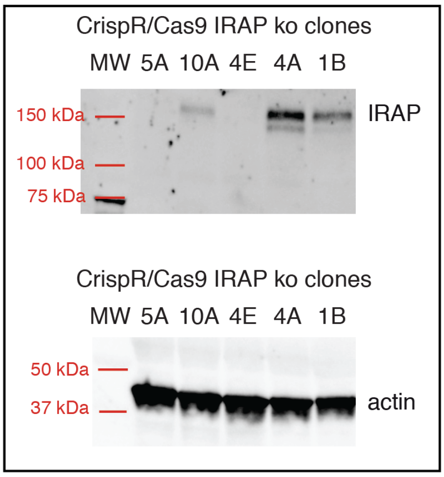

Supplementary Figure 1d

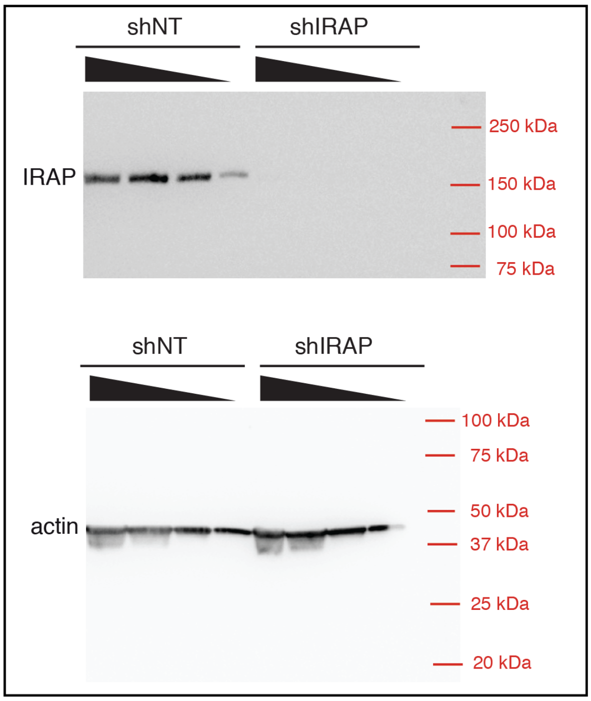

Supplementary Figure 1f

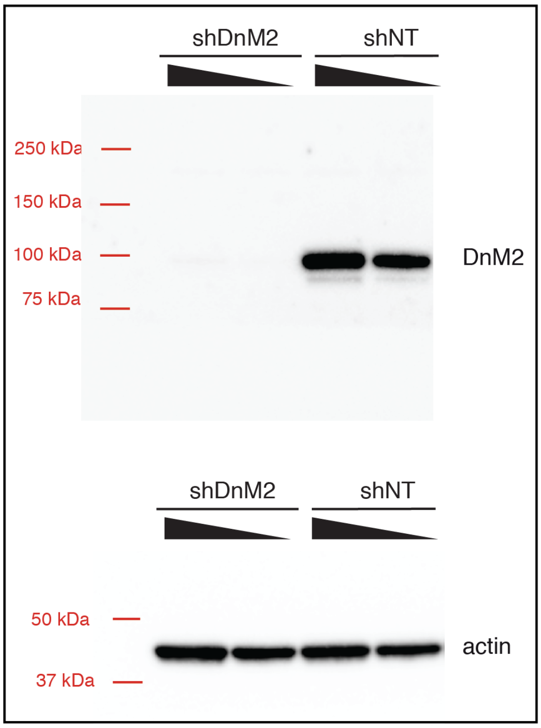

Supplementary Figure 9 Uncropped Original Scans

Supplementary Figure 2a

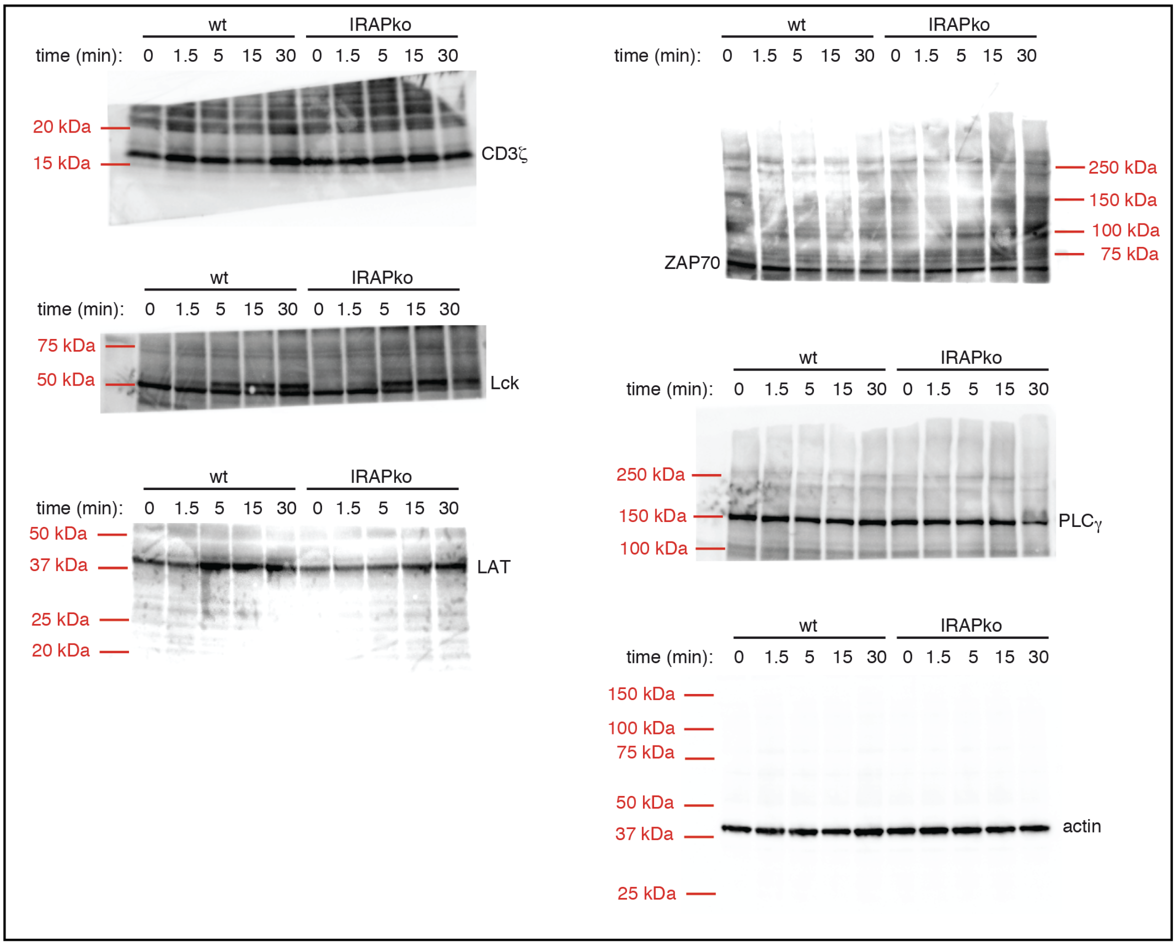

Supplementary Figures and Tables

Supplementary Table 1: Sequences of DNA primers used in the study:

For IRAP^{Tcellko} mice genotyping	
Lck-cre FW	5'-CGATGCAACGAGTGATGAGGTTC-3
Lck-cre REV	5'-GCACGTTCACCGGCATCAAC -3'
IRAPlox FW	5'-GATAAGATAGAAGTAGGGGAGA-3'
IRAPlox REV	5'-CAATAGAGGTACAGTCACCA-3
gKO	5'-GGAGAATAAGGGCTGTGAGAGA-3'
For CD8-CD3ζ cloning in pHR vector	
CD8-CD3ζ FW	5'-TCTCGAGAATTCTCACGCGTATGGCCTTACCAGTGACCG-3'
CD8-CD3ζ REV	5'-GATGATGGTGCCCTGGGTTAGCGAGGGGGCAGGG-3'
For mouse IRAP E465A cloning in pHR vector	
IRAP FW:	5'-TCTCGAGAATTCTCACGCGTGCCACCATGGAGTCCTTTACCA-3'
IRAP REV:	5'GATGATGGTGCCCTGGGCTAAGCGTAATCTGGAACATCGTATGGGT AGGCGTAGTCGGGCACGTC -3'
For mouse IRAP 3CA cloning in pHR vector	
IRAP FW:	5'TCTCGAGAATTCTCACGCGTGCCACCATGGAGTCCTTTACCAATGAT CGGCTT -3'
IRAP REV:	5'- GATGATGATGGTGCCCTGGGCTATCCCGACAGCCACTGG-3'
For CRISPR/Cas9 knock-out of IRAP	
sgIRAP FW:	5'-CACCGTTCATAGCGTAGTGGCACAA-3'
sgIRAP REV:	5'-AAACTTGTGCCACTACGCTATGAAC-3'
sgNT1	5'-GCGAGGTATTCGGCTCCGCG-3'
sgNT2	5'-GCTTTCACGGAGGTTCGACG-3'
For quantitative RT-PCRs	
AP2μ FW:	5'- TAAGATGTGTGACGTGATGG -3'
AP2μ REV:	5'- GATACTTGATACCCTCTCGC -3'
ACTIN-B FW:	5'- CTGGAACGGTGAAGGTGACA-3'
ACTIN-B REV:	5'- AAGGGACTTCCTGTAACAACGCA-3'
GAPDH FW:	5'- TGCACCACCAACTGCTTAGC-3'
GAPDH REV:	5'- GGCATGGACTGTGGTCATGAG-3'
For Rab4-GFP cloning	
Rab4b FW:	5'-TATTTCCGGTGAATTCATGGTGAGCAAGGGCGAGG-3'
Rab4b REV:	5'-TAGTCTCGAGGAATTCTCAGCAGCCACAGGGCTGG-3'
