# Supplementary material for: IRAP-dependent endosomal T cell receptor signalling is essential for T cell responses
Source: Nat Commun. 2020 Jun 2;11:2779. doi: 10.1038/s41467-020-16471-7 (PMC7265453; doi:10.1038/s41467-020-16471-7)
Supplement: Supplementary file 2 — Reporting Summary [file 41467_2020_16471_MOESM2_ESM.pdf]

nature research

Corresponding author(s): Loredana SAVEANU, Irini EVNOUCHIDOU

Last updated by author(s): Apr 22, 2020

Reporting Summary

Nature Research wishes to improve the reproducibility of the work that we publish. This form provides structure for consistency and transparency in reporting. For further information on Nature Research policies, see Authors & Referees and the Editorial Policy Checklist.

Statistics

For all statistical analyses, confirm that the following items are present in the figure legend, table legend, main text, or Methods section.

n/a	Confirmed	
☐	☒	The exact sample size (*n*) for each experimental group/condition, given as a discrete number and unit of measurement
☐	☒	A statement on whether measurements were taken from distinct samples or whether the same sample was measured repeatedly
☐	☒	The statistical test(s) used AND whether they are one- or two-sided<br>*Only common tests should be described solely by name; describe more complex techniques in the Methods section.*
☒	☐	A description of all covariates tested
☒	☐	A description of any assumptions or corrections, such as tests of normality and adjustment for multiple comparisons
☐	☒	A full description of the statistical parameters including central tendency (e.g. means) or other basic estimates (e.g. regression coefficient) AND variation (e.g. standard deviation) or associated estimates of uncertainty (e.g. confidence intervals)
☐	☒	For null hypothesis testing, the test statistic (e.g. *F*, *t*, *r*) with confidence intervals, effect sizes, degrees of freedom and *P* value noted<br>*Give P values as exact values whenever suitable.*
☒	☐	For Bayesian analysis, information on the choice of priors and Markov chain Monte Carlo settings
☒	☐	For hierarchical and complex designs, identification of the appropriate level for tests and full reporting of outcomes
☒	☐	Estimates of effect sizes (e.g. Cohen's *d*, Pearson's *r*), indicating how they were calculated

Our web collection on statistics for biologists contains articles on many of the points above.

Software and code

Policy information about availability of computer code

Data collection	FRET analysis tool- SymPhoTime 64 Software, Leica Application Suite X (LAS X) software, ZEN Black (ZEISS) software, FACSDiVa (Becton Dickinson) software, Magellan standard software (Tecan).
Data analysis	ImageJ 1.44, Fiji 2.0.0-rc-54/1.51h, GraphPad Prism 7, FlowJo 10.6.1, Image Lab 5.2.1. (Biorad), Imaris 8 (Bitplane).

For manuscripts utilizing custom algorithms or software that are central to the research but not yet described in published literature, software must be made available to editors/reviewers. We strongly encourage code deposition in a community repository (e.g. GitHub). See the Nature Research guidelines for submitting code & software for further information.

Data

Policy information about availability of data

All manuscripts must include a data availability statement. This statement should provide the following information, where applicable:

- Accession codes, unique identifiers, or web links for publicly available datasets
- A list of figures that have associated raw data
- A description of any restrictions on data availability

Full scans of the gels and blots are available in Supplementary Fig. 9. Raw data for all figures and supplementary figures are available as a source data file. All other data are included in the supplemental information or available from the authors upon reasonable requests.

Field-specific reporting

Please select the one below that is the best fit for your research. If you are not sure, read the appropriate sections before making your selection.

☒ Life sciences ☐ Behavioural & social sciences ☐ Ecological, evolutionary & environmental sciences

For a reference copy of the document with all sections, see nature.com/documents/nr-reporting-summary-flat.pdf

Life sciences study design

All studies must disclose on these points even when the disclosure is negative.

Sample size	The chosen sample size is based on the numbers used for previous publications, which is most optimal to generate statistically significant results. Animal sample size was determined by experimental feasibility and sample availability to demonstrate certain results.
Data exclusions	There was no data exclusion.
Replication	All experiments contained at least duplicates for each point. The majority of the experiments were performed at least 3 times. The experiments realized 2 times are shown in the Figures 1k; 2a (only the pZAP blot); 3d,e,f; 4f; 5a,b,c,d; 6e; 7b,c and Supplementary Figures 1a,b,e,g,h; 2b, 2c (righ panel), 2d; 4b; 5a, b, d; 6a, b, c, d. The experiment realized only 1 time is the screening of CRISPR/Cas9 IRAP ko clones by western blot (Supplementary Figure 1c), in which two IRAP ko clones were identified. The stability of these clones was verified by an additional western blot two months later and by immunofluorescence each month. The attempts at replication were successful in all the experiments shown.
Randomization	Mice were allocated to experimental groups according to their specific genotype or were randomly assigned into groups when the experiment involved mice of the same genotype.
Blinding	Blinding was not relevant to the study because all samples were analyzed in the same way.

Reporting for specific materials, systems and methods

We require information from authors about some types of materials, experimental systems and methods used in many studies. Here, indicate whether each material, system or method listed is relevant to your study. If you are not sure if a list item applies to your research, read the appropriate section before selecting a response.

Materials & experimental systems

n/a	Involved in the study	
☐	☒	Antibodies
☐	☒	Eukaryotic cell lines
☒	☐	Palaeontology
☐	☒	Animals and other organisms
☒	☐	Human research participants
☒	☐	Clinical data

Methods

n/a	Involved in the study	
☒	☐	ChIP-seq
☐	☒	Flow cytometry
☒	☐	MRI-based neuroimaging

Antibodies

Antibodies used

The following antibodies for FACS were from BIOLEGEND:
CD4-BV785 (GK1.5, #100453), CD44-PE-Cy7 (IM7, #103030), TCRb-APC (H57-597, #109212), CD8- PerCP-Cy5.5 (53-6.7, #100734), CD45-APC-Cy7 (30-F11, #103116), CD25-FITC (3C7, #101908), CD127-BV421 (A7R34, #135023), CD69-PE (H1.2F3, #104508) (for thymus samples only), CD62L-PE (MEL-14, #104407) (for spleen and lymph node samples only), CD5-A700 (53-7.3, #100636), I-A/I-E-biotin (M5/114.15.2, #107604), CD11b-FITC (M1/70, #101206), GR1-biotin (RB6-8C5, #108403), F4/80-biotin (BM8, #123105), CD45.1-APC-Cy7 (A20, #110716), CD45.2-A700 (104, #109822), R-PE labelled Pro5 MHC Pentamer H-2Kb SIINFEKL (#F093-2A) was from ProImmune. Anti-human CD3e (OKT3, #317326) was from Biolend and anti-human CD8a (OKT8, #14-0086-80) was from Thermo Fisher Scientific. Mouse anti-IRAP-AF594 (F-5, #sc-365300 AF594, Santa Cruz Biotechnology), rabbit anti-Stx6 (#10841-1-AP, ProteinTech) or monoclonal rabbit IgG (DA1E, #3900, Cell Signaling Technology) were used for FACS intracellular staining.
Antibodies used for immunoblots: rabbit anti-IRAP (homemade, provided by S. Keller, Virginia University, USA, published in "Keller, S. R., Scott, H. M., Mastick, C. C., Aebersold, R. & Lienhard, G. E. Cloning and Characterization of a Novel Insulin-regulated Membrane Aminopeptidase from Glut4 Vesicles. J. Biol. Chem. 270, 23612–23618 (1995)"), rabbit anti-IRAP (D7C5, #6918, Cell Signaling Technology), mouse anti-IRAP (3E1, #9876, Cell Signaling Technology), mouse anti-Lck (3A5, #sc-433, Santa Cruz Biotechnology), mouse anti-CD3ζ (6B.10.2, #sc-1239, Santa Cruz Biotechnology), mouse anti-β-actin (AC-15, #A5441, Sigma-Aldrich), rabbit anti-LAT (#06-807, Millipore), mouse anti-CD247 (pY142) (K25-407.69, #558402, BD Pharmingen), rabbit anti-pLAT (#3584S), rabbit anti-ZAP70 (D1C10E, #3165), rabbit anti-pZAP70 (65E4, #2717), rabbit anti-pPLCγ1 (#2821), rabbit anti-PLCγ1 (#2822) and rabbit antipSrc (D49G4, #6943) (all from Cell Signaling Technology). Rat anti-FLAG (L5, #637301) was from Biolegend and goat anti-mouse IgG (#115-035-146), anti-mouse IgG light chain (#115-035-174), anti-rabbit IgG (#111-035-144)

(all coupled with HRP) were from Jackson Immunoresearch.
Antibodies used for T cell activation: 125 ng/ml mouse anti-CD3ε (OKT3, #317326, Biolegend) and 250 ng/ml mouse anti-CD28 (CD28.2, #302934, Biolegend); 1 µg/ml hamster anti-CD3ε (145-2C11, #100340, Biolegend) and 2 µg/ml anti-CD28 (37.51, #102116, Biolegend); 1,33 µg/ml mouse anti-Armenian and Syrian hamster IgG1 (G9456, #554005, BD Biosciences).
Antibodies used for microscopy: mouse anti-CD3ε (OKT3, #317326, Biolegend), mouse anti-CD3ζ (6B.10.2, #sc-1239, Santa Cruz Biotechnology), Alexa-Fluor 488 mouse anti-CD247 (pY142) (K25-407.69, #558486, BD Biosciences), rabbit anti-calnexin (#C4731, Sigma-Aldrich), rabbit anti-LAMP1 (#L1418, Sigma-Aldrich), rabbit anti-Stx6 (#10841-1-AP, ProteinTech), rabbit anti-IRAP (homemade, a generous gift from S. Keller, Virginia University, USA), mouse anti-Lck (3A5, #sc-433, Santa Cruz Biotechnology), goat anti-EEA1 (#sc-6415, Santa Cruz Biotechnology), rabbit anti-DnM2 (#PA1-661, Thermo Fisher Scientific), rabbit anti-LAT (#06-807, Millipore), rabbit anti-Rab7 (H-50, #sc-10767, Santa Cruz Biotechnology), rat anti-LFA-1 (#553337, BD Biosciences), hamster anti-TCRb (H57-597, #553337, Biolegend), rabbit anti-IRAP (D7C5, #6918), mouse anti-IRAP (3E1, ##9876,), rabbit anti-ZAP70 (D1C10E, #3165), rabbit anti-pZAP70 (65E4, #2717) AF488 alpaca anti-mouse IgG1 (Chromotek, #sms1AF488) and rabbit anti-pSrc (D49G4, #6943) (all from Cell Signaling Technology).
Secondary antibodies coupled with Alexa fluorochromes were from Molecular Probes (Thermo Fisher Scientific): anti-mouse A488 (#A32766), A594 (#A32744), A647 (#A32787), anti-rabbit A405 (#A31556), A488 (#A32790), A594 (#A32754), A647 (#A32795).

Validation

All anti-IRAP antibodies used in this study (rabbit anti-IRAP from S. Keller, clones 3E1 and D7C5 from Cell Signalling Technology and clone F5 from Santa Cruz) were validated by us by immunoblot, FACS and immunofluorescence using IRAP deficient cells.
The following antibodies: rabbit anti-pLAT (#3584S), rabbit anti-ZAP70 (D1C10E, #3165), rabbit anti-pZAP70 (65E4, #2717), rabbit anti-pPLCγ1 (#2821), rabbit anti-PLCγ1 (#2822) and rabbit anti-pSrc (D49G4, #6943)) were validated by Cell Signaling Technology, as published on their website (https://www.cellsignal.com/contents/our-approach/cst-antibody-validation-principles/ ourapproach-validation-principles).
The rat anti-Flag tag (Clone L5, BioLegend, #637301) was validated by us using cell lysates that do not express the Flag tag.
The following antibodies: CD4-BV785 (GK1.5, #100453), CD44-PE-Cy7 (IM7, #103030), TCRb-APC (H57-597, #109212), CD8-PerCP-Cy5.5 (53-6.7, #100734), CD45-APC-Cy7 (30-F11, #103116), CD25-FITC (3C7, #101908), CD127-BV421 (A7R34, #135023), CD69-PE (H1.2F3, #104508), CD62L-PE (MEL-14, #104407), CD5-A700 (53-7.3, #100636), I-A/I-E-biotin (M5/114.15.2, #107604), CD11b-FITC (M1/70, #101206), GR1-biotin (RB6-8C5, #108403), F4/80-biotin (BM8, #123105), CD45.1-APC-Cy7 (A20, #110716), CD45.2-A700 (104, #109822), anti-human CD3e (OKT3, #317326) were validated by BioLegends, according with their antibody validation and quality control strategies (https://www.biolegend.com/en-us/reproducibility).
All secondary antibodies (HRP coupled and Alexa Fluor – coupled) used for immunobloting and immunofluorescence were validated by Jackson Immunoresearch (https://www.jacksonimmuno.com/secondary-antibody-resource/technical-tips/cross-adsorbed-and-cross-reactivity/) and Invitrogen (https://www.thermofisher.com/fr/fr/home/life-science/antibodies/invitrogen-antibody-validation.html), respectively. In addition, we routinely check for secondary antibodies cross-reactivity in our laboratory and we confirmed the specificity of the secondary antibodies used.
The following antibodies: mouse anti-CD3ζ (6B.10.2, #sc-1239), mouse anti-Lck (3A5, #sc-433), goat anti-EEA1 (#sc-6415) were produced by Santa Cruz Biotechnologies and validated by (for anti-Lck see: https://www.labome.com/gene/mouse/Lck-antibody.html; for anti-CD3z see: https://www.labome.com/product/Santa-Cruz-Biotechnology/sc-1239.html; for anti-EEA1 see: https://www.scbt.com/p/eea1-antibody-n-19).
AF488 alpaca anti-mouse IgG1 (#sms1AF488) was validated by Chromotek (https://www.chromotek.com/products/detail/ product-detail/alpaca-anti-mouse-igg1-recombinant-vhh/).
The rabbit anti-STX6 antibody (#10841-1-AP) was validated using knock-out cells by ProteinTech (https://www.ptglab.com/ products/STX6-Antibody-10841-1-AP.htm).

Eukaryotic cell lines

Policy information about cell lines

Cell line source(s)

Jurkat T cells were provided by Claire Hivroz, Curie Institut, Paris, validated by SSTR method and present 88% of homology with DSMZ Leibniz ACC 282. EG7-ova cells were published in "Moore, M. W., Carbone, F. R. & Bevan, M. J. Introduction of soluble protein into the class I pathway of antigen processing and presentation. Cell 54, 777–785 (1988)", Raji B cells were from ATCC (#CCL-86), DC2.4 were from Sigma Aldrich (#SCC142) and HEK293FT cells were from Invitrogen(# R70007). Daju-A2 cells published in "Moore, R. et al. Involvement of cadherins 7 and 20 in mouse embryogenesis and melanocyte transformation. Oncogene 23, 6726–6735 (2004)" were provided by Florence Faure, Curie Institut Paris and validated by FACS for HLA-A2 expression using the mouse anti-HLA-A2 antibody (clone BB7.2, BioLegend #343302).

Authentication

Only the Jurkat T cells were validated by SSTR method and present 88% of homology with DSMZ Leibniz ACC 282.

Mycoplasma contamination

Venor GeM Classic: Mycoplasma detection kit for conventional PCR -did not detect mycoplasma contamination.

Commonly misidentified lines
(See ICLAC register)

The cell lines Jurkat, Raji-B, Daju-A2, HEK293FT, DC2.4 are not found in the version 10 of ICLAC.

Animals and other organisms

Policy information about studies involving animals; ARRIVE guidelines recommended for reporting animal research

Laboratory animals

IRAPlox/lox, OT1, IRO, IRAP-deficient, IRAPTcellko and CD45.1 mice were on C57/BL6 background. We used both male and female animals between 6 and 12 weeks of age. Control mice were littermates and in all the experiments mice were matched for sexe and age.

Wild animals

No wild animals were used in the study.

Field-collected samples	No field collected samples were used in the study.
Ethics oversight	All animal experiments, including the tumour injections, were approved by the Comité d'éthique pour l'expérimentation animale Paris-Nord/No 121 (APAFIS #16488), additionally approved by the CRI U1149 Ethical Committee and by the French Committee for OGM (DUO n° 5643).

Note that full information on the approval of the study protocol must also be provided in the manuscript.

Flow Cytometry

Plots

Confirm that:

☒ The axis labels state the marker and fluorochrome used (e.g. CD4-FITC).

☒ The axis scales are clearly visible. Include numbers along axes only for bottom left plot of group (a 'group' is an analysis of identical markers).

☒ All plots are contour plots with outliers or pseudocolor plots.

☒ A numerical value for number of cells or percentage (with statistics) is provided.

Methodology

Sample preparation	For tumour infiltration:<br>Tumours were extracted, weighed and digested with collagenase D (Roche) and Dnase I (Thermo Fisher Scientific). After red blood cell lysis with RBC lysis buffer (Biolegend), cells were stained with Ghost Violet™ 510 Viability Dye (TONBO Biosciences) for dead cell exclusion and then with the R-PE labelled Pro5 MHC Pentamer H-2Kb SIINFEKL (ProImmune). Cells were then blocked with Fc block (2.4G2, BD Biosciences) to prevent non-specific binding and stained with the following anti-mouse antibodies: CD45-APC-Cy7 (30-F11), CD8-perCP-Cy5.5 (53-6.7), CD4-BV785 (GK1.5), CD44-PE-Cy7 (IM7), I-A/I-E-biotin (M5/114.15.2), CD11b-FITC (M1/70), GR1-biotin (RB6-8C5), F4/80-biotin (BM8). All antibodies were from Biolegend.<br>For T cell adoptive transfer experiment:<br>Cells from the lymph nodes, the spleen and the tumour were stained with anti-mouse CD45.1-APC-Cy7 (A-20), CD45.2-A700 (104), TCRb-APC (H57-597), CD8-PerCP-Cy5.5 (53-6.7), CD4-BV785 (GK1.5), CD44-PE-Cy7 (IM7), I-A/I-E-biotin (M5/114.15.2), CD11b-FITC (M1/70), GR1-biotin (RB6-8C5), F4/80-biotin (BM8) and 7-AAD for dead cell exclusion. All antibodies were from Biolegend. AccuCheck counting beads were added to each sample in order to calculate absolute cell numbers.<br>For mouse phenotyping:<br>Cells were stained with Ghost Violet™ 510 Viability Dye (TONBO Biosciences) for dead cell exclusion, blocked with Fc block (2.4G2, BD Biosciences) and stained with the following anti-mouse antibodies from Biolegend: CD4-BV785 (GK1.5), CD44-PE-Cy7 (IM7), TCRb-APC (H57-597), CD8-PerCP-Cy5.5 (53-6.7), CD45-APC-Cy7 (30-F11), CD25-FITC (3C7), CD5-A700 (53-7.3), CD127-BV421 (A7R34) and CD69-PE (H1.2F3) (for thymus samples only) or CD62L-PE (MEL-14) (for spleen and lymph node samples only). AccuCheck counting beads were added to each sample in order to calculate absolute cell numbers.
Instrument	Samples were analysed on a Fortessa (BD Biosciences) instrument. For sorting, cells were sorted on a BD FACSMelody Cell Sorter or a SONY SH800 sorter (Sony Biotechnology).
Software	FACSDiva (BD Biosciences) software was used for data acquisition and FlowJo software for data analysis.
Cell population abundance	Cell purity was >99% determined by post-sort purity checks of representative samples.
Gating strategy	Cells were pregated on leucocytes by FSC-A vs SSC-A and doublets were excluded by FSC-A vs FSC-H.<br>For steady state T cell subpopulations: In the lymph nodes and spleen: TCRb+ cells were gated on live CD45+ cells. Within the TCRb+ gate, cells were further classified as CD4+ or CD8+. In the thymus: After gating on live CD45+ cells, cells were classified as DN, DP, CD4 SP and CD8 SP. The DP subset was further divided in DP1, DP2 and DP3 cells. The DN population was more precisely gated as negative for TCRb (real DN) and was further divided in DN1, DN2, DN3 and DN4 cells.<br>For T cell subpopulations in EG7-ova tumour: For wt or IRAPTcellko mice: Within the live CD45+ cells, cells were further classified as CD4+ or CD8+ after exclusion of (CD11b, GR-1, F4/80 and I-A/I-E)+ cells. CD44+-KbN4-tetramer+ cells were gated inside the CD8+ population. For adoptive transfer experiment: TCRb+CD8+ cells were gated on live-(CD11b, GR-1, F4/80 and I-A/I-E)- cells. OT1 or IRO injected cells were recognized as CD45.2+ whereas host cells were CD45.1+ (or CD45.1.2+ in some cases).

☒ Tick this box to confirm that a figure exemplifying the gating strategy is provided in the Supplementary Information.
