# Supplementary material for: IRAP-dependent endosomal T cell receptor signalling is essential for T cell responses
Source: Nat Commun. 2020 Jun 2;11:2779. doi: 10.1038/s41467-020-16471-7 (PMC7265453; doi:10.1038/s41467-020-16471-7)
Supplement: Supplementary file 3 — Description of Additional Supplementary Files [file 41467_2020_16471_MOESM3_ESM.pdf]

Description of Additional Supplementary Files

Supplementary Movies 1 and 2:

CD3ζ recruitment to the IS of wt Jurkat T cells. SEE-loaded Raji cells were seeded on IbiTreat microscopy chambers. Wt Jurkat T cells expressing CD3ζ-GFP were added to Raji cells under the microscope and the formation of T cell-Raji conjugates was recorded immediately. Time is in minutes, as indicated in the movie. Raji cells were labelled with CTV and are blue. Movies are representative for two independent experiments.

Supplementary Movies 3 and 4:

CD3ζ recruitment to the IS of IRAP deficient Jurkat T cells. SEE-loaded Raji cells were seeded on IbiTreat microscopy chambers. IRAP ko Jurkat T cells expressing CD3ζ-GFP were added to Raji cells under the microscope and the formation of T cell-Raji conjugates was recorded immediately. Time is in minutes, as indicated in the movie. Both Jurkat and Raji cells were labelled with CTV and are blue. Movies are representative for two independent experiments.
